# An integrated host-microbiome response to atrazine exposure mediates toxicity in *Drosophila*

**DOI:** 10.1038/s42003-021-02847-y

**Published:** 2021-11-24

**Authors:** James B. Brown, Sasha A. Langley, Antoine M. Snijders, Kenneth H. Wan, Siti Nur Sarah Morris, Benjamin W. Booth, William W. Fisher, Ann S. Hammonds, Soo Park, Richard Weiszmann, Charles Yu, Jennifer A. Kirwan, Ralf J. M. Weber, Mark R. Viant, Jian-Hua Mao, Susan E. Celniker

**Affiliations:** 1 Environmental Genomics and Systems Biology Division, Lawrence Berkeley National Laboratory, Berkeley, CA 94720 USA; 2 Centre for Computational Biology, University of Birmingham, Edgbaston, Birmingham, B15 2TT UK; 3 Department of Statistics, University of California Berkeley, Berkeley, CA 94720 USA; 4 Arva Intelligence, Inc, Salt Lake City, UT 84101 USA; 5 Biological Systems and Engineering Division, Lawrence Berkeley National Laboratory, Berkeley, CA 94720 USA; 6 School of Biosciences, University of Birmingham, Edgbaston, Birmingham, B15 2TT UK; 7 Present Address: Genentech Inc., 1 DNA Way, South San Francisco, CA 94080 USA; 8 Present Address: Berlin Institute of Health@Charité, Anna-Louisa-Karsch-Str. 2, DE 10178 Berlin, Germany

**Keywords:** Transcriptomics, Microbiome, Metabolomics

## Abstract

The gut microbiome produces vitamins, nutrients, and neurotransmitters, and helps to modulate the host immune system—and also plays a major role in the metabolism of many exogenous compounds, including drugs and chemical toxicants. However, the extent to which specific microbial species or communities modulate hazard upon exposure to chemicals remains largely opaque. Focusing on the effects of collateral dietary exposure to the widely used herbicide atrazine, we applied integrated omics and phenotypic screening to assess the role of the gut microbiome in modulating host resilience in *Drosophila melanogaster*. Transcriptional and metabolic responses to these compounds are sex-specific and depend strongly on the presence of the commensal microbiome. Sequencing the genomes of all abundant microbes in the fly gut revealed an enzymatic pathway responsible for atrazine detoxification unique to *Acetobacter tropicalis*. We find that *Acetobacter tropicalis* alone, in gnotobiotic animals, is sufficient to rescue increased atrazine toxicity to wild-type, conventionally reared levels. This work points toward the derivation of biotic strategies to improve host resilience to environmental chemical exposures, and illustrates the power of integrative omics to identify pathways responsible for adverse health outcomes.

## Abstract (web summary)

Brown et al. apply integrated omics and phenotypic screening to assess the role of the gut microbiome in modulating host resilience in Drosophila melanogaster. They find that Acetobacter tropicalis in gnotobiotic animals, is sufficient to rescue increased atrazine toxicity, which could pave the way for biotic strategies to improve host resilience to environmental chemical exposure.

## Introduction

Pesticides include herbicides, fungicides, and insecticides, and are applied in excess of 5.6 billion pounds each year^1^. The resulting ecological distributions of these chemicals give rise to a global health challenge—the World Health Organization estimates that 25 million individuals are hospitalized each year with pesticide-induced maladies^1^. In the US, these illnesses cost over $1.2B dollars in health care alone^2^. Thirty-three million Americans drink atrazine-contaminated water, and more than fifty million experience chronic low-dose exposure to one or more widely used pesticides^3^. During the next 30 years, agricultural productivity will need to increase by over 50% to feed our growing global population^4^, and similarly increasing energy demands met in part by biofuels^5,6^, will lead to a concomitant scale-up of the environmental distributions of pesticides world-wide.

Remediation strategies for the ecological and human health effects of pesticide usage focus on preventing exposure, but this is challenging given the scale of the problem, and the substantial benefits associated with pesticide use—i.e., the feasibility of supplying fresh produce to our growing population^4^. It is clear that humans exhibit substantial individual variability in responses to pesticide exposure, but the source of variation is poorly understood^7^. Polymorphisms in P450 cytochromes have been associated with increased risk for adverse health outcomes from low dose exposure for particular compounds^8,9^. Many pesticides, such as the triazine herbicide atrazine, are not effectively transformed by metazoan metabolisms, but are rapidly detoxified by microbes^10^. In some pests, it has been shown that the host gut microbiome plays a role in establishing individual susceptibility to insecticides exposure by modulating effective dosage in the gut compartment^11–13^. However, few such studies have been conducted, and the capacity of commensal microbial consortia to modulate toxicity through chemical transformation requires additional exploration and detailed case studies.

The gut microbiome plays a major role in the metabolism of xenobiotic compounds, including the pharmacokinetics of many drugs^13–17^. Studies in tractable genetic model systems have revealed complex host-microbiome interactions^18^. Here, we used the genetic model organism, *Drosophila melanogaster*, to identify microbes that participate in adaptive responses to chronic pesticide exposure in aerobic and anaerobic conditions. We investigated host-microbiome interactions using gnotobiotic studies and multi-omics measurements, including RNA sequencing, 16S profiling, metagenomics, and metabolomics, to identify co-responsive genes, microbes, and molecules associated with adverse host phenotypes. Ultimately, understanding the role of the gut microbiome in adaptation to chronic pesticide exposure may lead to novel therapeutic strategies surrounding the application of pre- and pro-biotics with the potential to improve the health of tens of millions of people worldwide and improve the resilience of keystone pollinators, such as flies and bees, to pesticide-induced population collapse.

## Results

### Drosophila as a highly tractable exposure model

**Fig. 1:**
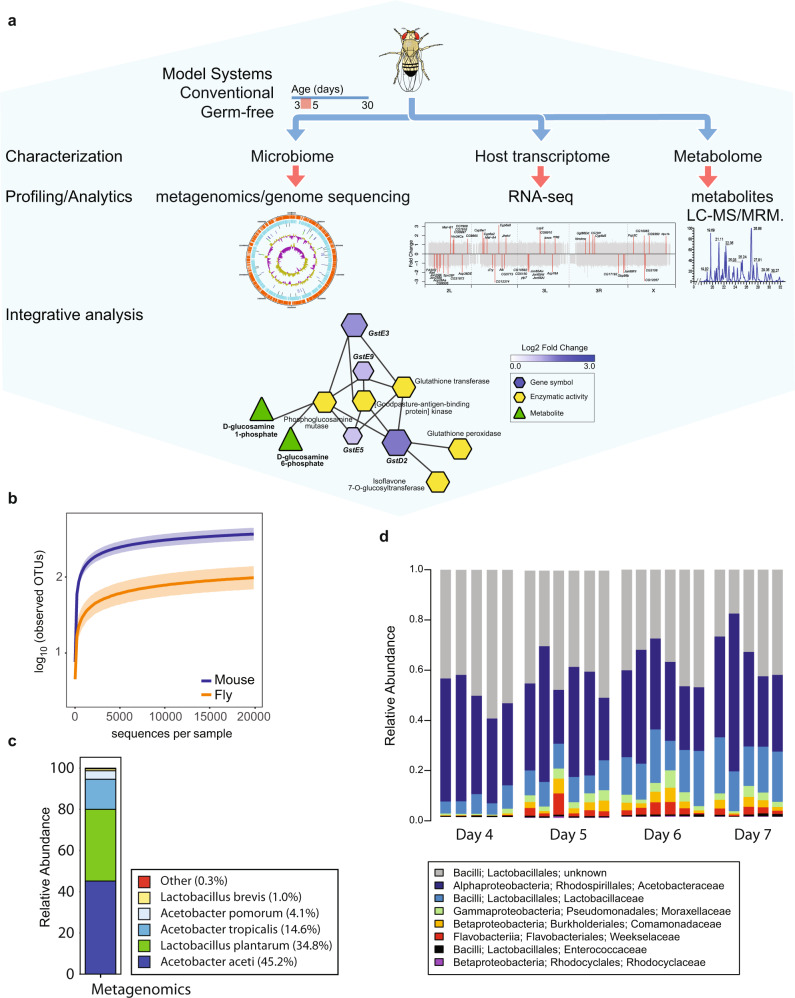
Baseline microbiome composition in *Drosophila melanogaster*. **a** Study design. **b** Rarefaction curves of the observed OTUs to assess species richness in *Drosophila* (orange; obtained from AdMMF at 4−8 days of age) and *Mus musculus* (purple; obtained from C57BL/6J fecal samples at 12 weeks of age). **c** Metagenomic sequence analysis to determine the bacterial species composition of the Drosophila gut microbiome isolated from 20 adult females at 21 days of age. The bar indicates relative abundance level colored at the species level as indicated in the key. **d** Age-dependent change in the distribution of the *Drosophila* gut microbiome based on 16S sequencing of fecal samples. For each of the 16S experiments, embryos were distributed between 25 bottles on chemically defined fly food. Four days post-eclosion flies were collected and the weight-equivalent of 250 flies were transferred to small cages (100 mm diameter × 150 mm) for the aging study. The food was replaced once daily. Bars indicate relative bacterial abundance colored at the family level as indicated in the key.

We designed a multi-omic study to investigate the role of the microbiome on pesticide metabolism using *Drosophila melanogaster* as a model system (Fig. 1a). We exposed conventionally reared four-day-old flies to atrazine or paraquat for three consecutive days. To identify, classify and determine the microbial community composition of the fly microbiome, we collected fecal swabs for 16S ribosomal RNA profiling and metabolomics analysis. To monitor the effects of herbicide exposure on the host, we performed RNA-seq analysis of whole flies.

Environmental and food-born microbes rapidly colonize the adult fly gut shortly after eclosion. A number of studies have characterized laboratory and natural populations, and there is substantial variation across strains and environmental conditions. Therefore, prior to starting our exposure studies, we first characterized the gut microbiome of Oregon-R-modENCODE flies in our laboratory environment. Relative to the mouse microbiome we observed approximately 100-fold fewer species in the fly gut, consistent with previous reports (Fig. 1b; Supplementary Data 1 and 2). The fly microbiome was dominated by two phyla: *Firmicutes* and *Proteobacteria*; and two genera: *Lactobacillus* and *Acetobacter* (Fig. 1c; Supplementary Data 1 and 2). Over time, the relative abundances changed and the microbiome became more complex with an increase in the relative abundance of initially rare species (Fig. 1d) consistent with previous studies^19,20^.

**Fig. 2:**
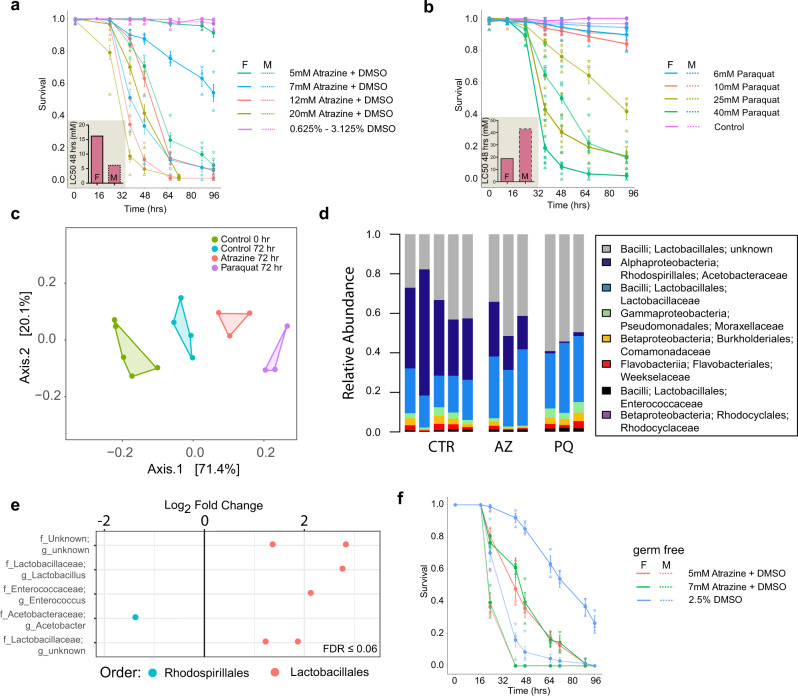
Effect of atrazine and paraquat on survival and gut microbiome in *Drosophila melanogaster*. Effects of exposures on host and microbiome. **a** Host survival curves in response to 5−20 mM atrazine exposures. LC50 at 48 h is 16 mM for females and 7 mM for males. For each dose, we used 30 males and 30 females in triplicate aged 4−5 days post eclosion. Error bars indicate the standard deviation across replicates. **b** Host survival curves in response to 6−40 mM paraquat exposures. LC50 at 48 h is 19 mM for females and 43 mM for males. For each dose, we used 30 males and 30 females in triplicate aged 4−5 days post eclosion. Error bars indicate the standard deviation across replicates. **c** Principal component analysis of microbial abundance levels in control, atrazine, and paraquat treated flies. The control 0 and 72 h groups are closer to each other than the atrazine and paraquat groups at 72 h. **d** Bar charts showing the relative abundance of bacterial families in control (*n* = 5), 2 mM atrazine treated (*n* = 3) or 8 mM paraquat treated (*n* = 3) flies at 72 h after treatment. response to atrazine and paraquat. Both herbicides show a reduction in the amount of the Rhodospirillales grp and an increase in the Lactobacillales group. **e** Log_2_fold change at the genus level for bacteria significantly changed after treatment with atrazine (FDR < 0.06). Bacteria are color-coded at the order level. **f** Germ-free host survival curves in response to 5 and 7 mM atrazine exposure. For each dose, we used 30 males and 30 females in triplicate aged 4−5 days post eclosion. Error bars indicate the standard deviation across replicates.

We exposed adult flies (Oregon-R-modENCODE; BDSC strain 25211) to two commonly used herbicides: paraquat and atrazine, in chronic, collateral dietary scenarios lasting three to four days (72−96 h, 5% adult lifespan). Each herbicide was added to fly medium at a fixed concentration. First, we determined the LC50 for each herbicide by exposing adult flies to different doses ranging from 5 to 20 mM atrazine and 6 to 40 mM paraquat. These doses are commensurate with field-proximal exposures expected for pollinators, including *Diptera*^21^. Atrazine was solubilized in DMSO and paraquat in water. DMSO and water were used as controls for atrazine and paraquat exposures, respectively. Interestingly, we observed sex differences in sensitivity to both atrazine and paraquat (Fig. 2a, b). The LC50, defined as the 50% lethal concentration at 48 h, for atrazine was significantly different between males (5 mM) and females (12 mM) (Fig. 2a). In contrast, the females (LC50 = 20 mM) were more sensitive to paraquat exposures compared to males (LC50 = 43 mM) (Fig. 2b).

#### Microbiome remodeling under pesticide exposure

To determine the impact of herbicide exposure on the gut microbiome, fecal swabs were collected for 16S sequencing every 24 h during the 72 h atrazine and paraquat exposures. Each herbicide resulted in alterations to the microbiome composition (Fig. 2c, d; Supplementary Data 1 and 2)—intriguingly both reduced the relative abundance specifically of *Acetobacter* relative to *Lactobacillus* and other genera. Dietary paraquat exposure resulted in a 33-fold reduction in the relative abundance of all species and strains of *Acetobacter* by 72 h (Fig. 2d, e). Atrazine also impacted *Acetobacter*, reducing overall population abundance by two-fold (Mann−Whitney U test; *p* = 0.036) (Fig. 2d, e). For both atrazine and paraquat, we observed a robust increased abundance of species belonging to the order *Lactobacillales* and a decrease in abundance level of the order *Rhodospirillales* (Fig. 2e). These data show that pesticide exposure impacts specific genera of the gut microbiome. Whether these perturbations are accomplished through the direct action of the herbicide on the microbes or through modulation of host metabolism remains unclear.

Since atrazine is widely used in the US and its effect on terrestrial metazoans is less studied than paraquat, we focused our studies on the effects of atrazine on the host and microbiome. All data are submitted to public repositories as a resource (Supplementary Data 1).

To investigate the role of the microbiome on the LC50 after atrazine exposure, we conducted a dose-response analysis on germ-free reared (GF) adult flies. Similar to conventionally reared (CR) and exposed flies, male germ-free flies were more sensitive to atrazine compared to female flies (Fig. 2f). Interestingly, significant toxicity was observed for DMSO alone (2.5%) in germ-free flies, which was not observed in conventional flies (Fig. 2f). Based on this observation we reduced the DMSO concentration 10-fold to 0.25% for subsequent germ-free fly experiments.

#### Transcriptional responses to pesticide exposure

To investigate the transcriptional response to atrazine we performed whole animal RNA sequencing analysis of conventionally and germ-free reared flies before and after atrazine exposure.

We first studied the differences in gene expression between unexposed CR and GF flies. We found 38 genes in males and 23 genes in females (Supplementary Fig. 1 and Supplementary Data 3 and 4) showing differential gene expression FC ≥ 3, *p* ≤ 1E−06. The genes that show common males and female differential gene expression include the P450 Cytochromes, *Cyp6a16, Cyp304a1*, and *eye transformer* (*et)* that are activated in the absence of a microbiome, and *PGRP-SC1a and 1b, pirk* and *CG9759* that are repressed. *Cyp6a16* and *Cyp304a1* are likely associated with the detoxification of bioactive compounds. The gene *et* is a negative regulator of JAK/STAT signaling that is induced by and protective against bacterial infection^22^—why it is induced in the absence of a microbiome is not immediately apparent. *Peptidoglycan recognition protein SC1 a* and *b (PGRP-SC1a and b)* recognize and degrade bacterial cell wall structures, and *pirk* is a negative regulator of similar peptidoglycan recognition proteins. The function of gene *CG9759* remains unknown and requires further investigation.

It is well known that germ-free animals show altered physiology, an adaptation to the absence of microbes that includes reduced metabolism and changes in immunity. Overall, we see an increased expression in genes encoding proteins with serine-type endopeptidase, oxidoreductase, alkaline phosphatase, ammonium transmembrane transporter, cytokine receptor, gamma-butyrobetaine dioxygenase, and metalloendopeptidase activity (Fig S1b). We see a decreased expression in genes encoding proteins involved in peptidoglycan (the primary amino acid sugar component of bacterial cell walls) binding and degradation – consistent with the expression of bacteria-regulating proteins only in the presence of bacteria (Fig S1b).

**Fig. 3:**
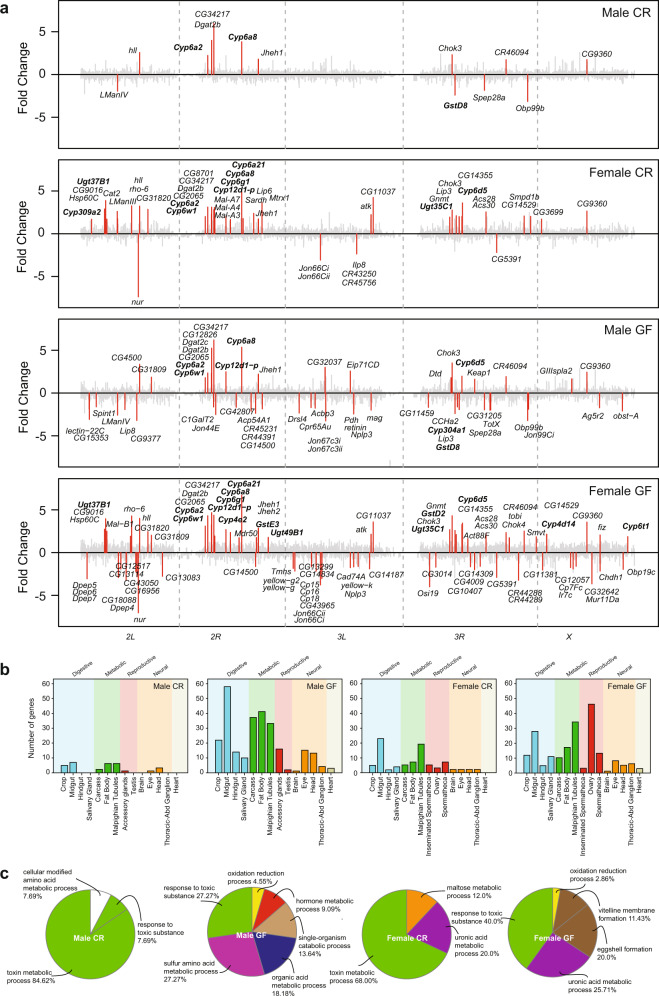
Effect of atrazine on host transcription in adult flies. Transcriptional profiling of males and females raised conventionally or germ-free after exposure to atrazine. **a** Genes with differential expression after 72 h atrazine exposure with fold changes ≥ 3 (adjusted *p* ≤ 1E−06) (red). Conventionally reared (CR) germ-Free reared (GF). Genes are ordered on the chromosomes X, 2L, 2, 3L, 3R, and 4. All genes are shown in gray. **b** Anatomical analysis of the genes identified by DeSeq2 (**a**) using their organ-specific maximal gene expression to assign each gene to a single organ system. **c** Gene Ontology analysis using ClueGO in Cytoscape (*p* < 0.05) of genes differentially expressed (FC ≥ 1.5 and adjusted *p* ≤ 1E−06) after 72 h in 2.0 mM atrazine treated CR and GF flies compared to CR and GF untreated control flies, respectively.

In males, 72 h after 2.0 mM atrazine exposure, we observed 33 genes at FC ≥ 1.5, adjusted *p* ≤ 1E−06 and 13 genes at FC ≥ 3, adjusted *p* ≤ 1E−06 (Fig. 3a; Supplementary Data 5). In females, we observed an increase in the number of deregulated genes compared to males in response to 2.0 mM atrazine exposure (92 genes at FC ≥ 1.5, adjusted *p* ≤ 1E−06; 47 genes at FC ≥ 3, adjusted *p* ≤ 1E−06) (Fig. 3a; Supplementary Data 6). Sixteen genes (FC ≥ 1.5, adjusted *p* ≤ 1E−06) were found deregulated after atrazine exposure in both males and females, which included *Cyp6a2, Cyp6a8*, and *Cyp6d5*, involved in response to toxic substances (*p* < 0.01). In contrast to CR flies, the transcriptional response to atrazine was more pronounced in GF flies. In germ-free treated male flies, we observed a eight-fold greater transcriptional response compared to CR treated male flies as evidenced by 274 genes with altered expression (274 genes at FC ≥ 1.5, adjusted *p* ≤ 1E−06; 55 genes at FC ≥ 3, adjusted *p* ≤ 1E−06) (Supplementary Data 7), whereas in treated female flies we observed only a two-fold greater transcriptional response compared to CR treated female flies, corresponding to 207 genes with altered expression (207 genes at FC ≥ 1.5, adjusted *p* ≤ 1E−06; 86 genes at FC ≥ 3, adjusted *p* ≤ 1E−06) (Supplementary Data 8). The transcriptional response of the majority of genes found in CR flies also responded in GF flies (32 out of 33 in males and 60 out of 92 in females)—though we observe intriguing differences: in male CR flies, *Glutathione S-Transferase D8* is down-regulated ~2-fold, whereas it shows no response in GF animals. More broadly, in comparison to CR flies, GF male and female flies show strong enrichment for genes involved in oxidation-reduction in response to atrazine exposure. In female GF flies, we found strong downregulation of reproductive genes involved in vitelline membrane (including *Vm26Aa*, *Vm26Ab, Vm32E,* and *psd*) and eggshell (including *Cad74a, Cp16, Cp7Fb, Cp7Fc,* and *Mur11Da*) formation (*p* < 0.01) indicating that atrazine exposure affects female fecundity (Fig. 3b). In male GF flies, genes involved in hormone, lipid, and organic acid metabolism were affected by atrazine exposure (*p* < 0.01) (Fig. 3c). In addition, similar to female GF flies, genes involved in the male reproductive system including *Acp54a1, Acp24A4* were downregulated. None of the genes that show differential gene expression in the CR versus GF flies were found in common with the treated samples except for a female repressed gene, *Jon66cii*, a chymotrypsin-like serine protease (Fig. 3c).

#### Acetobacter tropicalis rescues atrazine induced toxicity

**Fig. 4:**
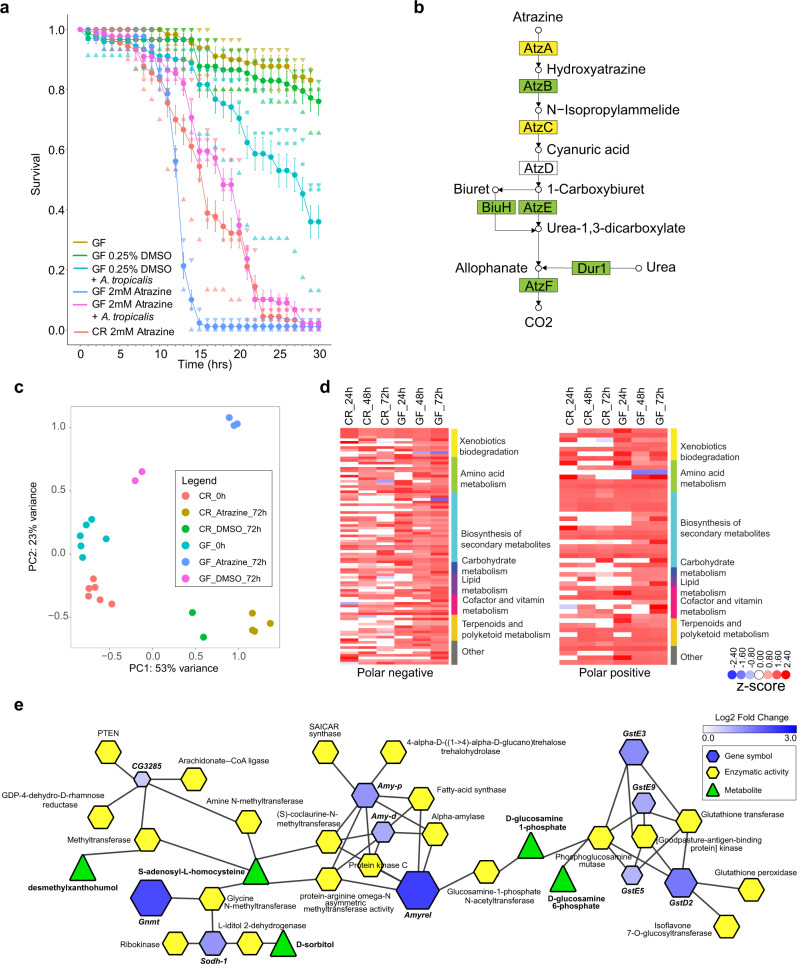
*Acetobacter tropicalis* partially rescues atrazine toxicity in germ-free flies. **a** Survival curves of *AdMMF* GF flies exposed to 2 mM atrazine, 2 mM atrazine supplemented with *A.tropicalis*. Survival curves of untreated GF, 0.25% DMSO treated GF, and 2 mM atrazine treated CR flies are included as controls. All atrazine treated flies die by 15 days whereas those supplemented with *Acetobacter tropicalis* survive for at least 23 days following the curve of flies reared conventionally. For each condition, we used 15 males and 15 females in triplicate aged 4−5 days post eclosion. Error bars indicate the standard deviation of the proportion of surviving flies. **b** Atrazine degradation pathway. *Acetobacter tropicalis* genes in green are those with high similarity to genes found on a *Pseudomonas* sp. strain ADP, pADP-1 plasmid (LKAX01000023), AtzE (ATJ92156.1), BiuH (ATJ90877.1) AtzE (ATJ91605.1), and AtzF (ATJ90896.1) Dur1 (ATJ90895.1); genes in yellow, AtzA and AtzC have weak similarity to a number of putative candidate genes including N-ethylammeline chlorohydrolase (ATJ89361.1) and D-glutamate deacylase (ATJ89456.1). *atzD* is the only gene not having any putative orthologs in the atrazine degradation pathway; **c** principal component analysis of metabolic features detected in CR and GF flies at zero or 72 h after atrazine treatment. **d** Heatmap of putatively annotated metabolites clustered and grouped by KEGG pathway classifications. Note that z-scores correspond to the inverse normal transform of ranks, not a measure of significance—*z*-scores are used to improve visualization only. **e** FlyScape visualization of the metabolomics data in the context of metabolic reactions (24, 48, or 72 h) and transcriptional changes in adult female flies (72 h timepoint; adjusted *p* ≤ 0.01). The network includes only putatively annotated metabolites and genes identified in our analyses.

GF flies showed increased sensitivity to atrazine compared to CR flies. To assess whether specific microbes could rescue atrazine-induced toxicity, we first characterized the microbiome at the species level. We performed metagenomic sequencing and found that the fly gut microbiome contains: *Lactobacillus plantarum* (34.8%), *L. brevis* (1.0%) and *Acetobacter aceti* (45.2%), *A. tropicalis* (14.6%), and *A. pomorum* (4.1%) (Fig. 1d). These five species were isolated and sequenced to generate complete genomes (Supplementary Figs. 2−7)^23–27^. Finally, we cultured GF adult mixed male and female flies (AdMMF) with *Acetobacter tropicalis* or *Lactobacillus brevis*—two species observed in the fly gut microbiome. We used AdMMF flies for this experiment to avoid confounding behavioral effects of single-sex housing. Interestingly, we observed that inoculation of DMSO treated GF flies with *A. tropicalis* reduced survival in comparison to DMSO treated GF flies (Fig. 4a). This finding suggests a potential interaction between *A. tropicalis* and DMSO that is more toxic though such a mechanism is unknown. Importantly, restoration of the gut microbiome with *A. tropicalis* reduced atrazine toxicity to the same level as toxicity observed in CR flies (Fig. 4a), whereas *L. mesenteroides* or *L. brevis* did not (Supplementary Fig. 8a, b). To identify candidate genes involved in atrazine metabolism that might be present in *A. tropicalis*, we searched for genes that are similar to genes involved in a well-known atrazine metabolizing pathway^28^. We found that candidate genes *atzA, atzB,* and *atzC* were present in *A. tropicalis* (Fig. 4b), but not in *L. brevis*, or any other species sequenced as part of this study (Supplementary Data 2 and Supplementary Figs. 2−7).

#### Metabolic responses to pesticide exposure

To assess the effects of these exposures on fly metabolism, we studied the fecal metabolome. Feces capture information about both host and microbiome metabolism, of potential importance for understanding toxicity. We used nanoelectrospray ionization (nESI) direct-infusion mass spectrometry (DIMS) to detect metabolites and lipids in each sample (Supplementary Data 9)^29^. We detected more than 6500 metabolic features occurring in at least 80% of samples. To study metabolic features present only in one condition, e.g., GF versus CR, we lowered our threshold on presence as follows: we retained all metabolic features present in at least 2/3rds (66%) of samples within a condition and then imputed missing values as previously described^29^. As we observed with transcriptomics, we see an exaggerated metabolic response in GF flies. Differential abundance analysis reveals that after 72 h 2975 metabolic features are modulated under atrazine exposure in CR flies, while 3297 metabolic features change in GF (see “Methods” and Supplementary Data 10).

The effect of exposure to atrazine, and indeed DMSO, dominates the variance we observe in the fecal metabolome (Fig. 4c). In principal component analysis, the first component (53% of variance) is dominated almost entirely by exposure status. Samples segregate by microbiome status in the second principal component (23% of variance). We examined differentially abundant metabolic features that correspond to specific KEGG identifiers (and assigned to pathways) and found that the majority of such metabolites correspond to secondary metabolism, the biodegradation of xenobiotic compounds, and terpenoid and polyketoid metabolism (Fig. 4d and Supplementary Data 9)^30^. Remarkably, none of the metabolic features observed that change under atrazine exposure are specific to CR animals, indicating that we have identified host, as opposed to microbial metabolites—or at least metabolites that are not unique to microbes.

We note that nearly all metabolic features that significantly change in abundance upon atrazine exposure to which we were able to assign KEGG identifiers increase, with only a few decreasing. This pattern does not hold for metabolic features in general (those to which we are unable to assign KEGG identifiers), in both GF and CR animals, about half of exposure-modulated metabolic features increase in abundance (and the other half decrease, Supplementary Data 10). The source of this apparent annotation bias is not immediately clear.

Unsurprisingly, glutathione metabolism is significantly represented in both metabolomics and transcriptomics data^31^. However, we note that while glutathione precursors and metabolic byproducts are modulated by atrazine exposure, glutathione itself is not—levels are remarkably steady in both CR and GF flies. Hence, while the metabolic flux of this key antioxidant in secondary metabolism changes, the active metabolite is effectively maintained at basal levels suggesting effective homeostasis (Fig. 4e).

## Discussion

Multi-omic interrogation of atrazine response in *Drosophila melanogaster* revealed correlated transcriptional, metabolic, and microbial changes over the course of 72 h after exposure. We found that the gut microbiome evolves dynamically during early adult life under conventional rearing conditions, along consistent trajectories. In flies, *Lactobacillus* and *Acetobacter* dominate the gut microbiome^19,32^. We found that dietary exposure to both paraquat and atrazine remodel the Drosophila gut microbiome, reducing the abundance of *Acetobacter* relative to other clades.

The host transcriptome also undergoes changes upon atrazine exposure—and gene expression changes are of larger magnitude and more numerous (more genes affected) in gnotobiotic animals as compared to those conventionally reared. Among the genes that change most significantly upon atrazine exposure in gnotobiotic animals are those that are already induced or repressed compared to CR animals in the absence of pesticide exposure. Some genes, e.g., that respond in CR animals are non-responsive in animals that lack a gut microbiome, e.g., *Glutathione S-transferase D8* is downregulated ~2-fold in CR versus unchanged in GF flies. However, over-all, genes involved in oxidation-reduction are more strongly induced in GF flies compared to CR in response to atrazine exposure.

There were also intriguing sex-specific effects, with males exhibiting broadly stronger transcriptional modulation compared to females, despite the fact that females generally consume more food^33,34^, and therefore likely more atrazine, than males.

Study of the fecal metabolome revealed that, in contrast to the host transcriptome, where only dozens of genes are significantly modulated in their expression levels, the metabolome undergoes profound quantitative changes, with more than a quarter of detectable mass features changing significantly. Intriguingly, despite the central importance of glutathione in response to oxidative stress, and the modulations of numerous genes and metabolites involved in glutathione cycling, glutathione itself remains in homeostasis upon exposure.

We found that GF flies exhibited more pronounced lethality than CR flies at the same dosage of atrazine. An explanation for this difference is the presence of an atrazine degradation pathway in *Acetobacter tropicalis*. Indeed, we found that gut colonization of *Acetobacter tropicalis* in gnotobiotic animals partially rescues lethality due to atrazine exposure. However, we cannot exclude the possibility that some atrazine degradation by *A. tropicalis* may occur in the fly media. Measurements of atrazine metabolites in bacteria medium in the absence or presence of *A. tropicalis* showed no significant difference in degradation suggesting that *A. tropicalis* alone is not sufficient in degrading atrazine (Supplementary Fig. 8c). Although our study used a food dye to exclude starvation as a confounder, we did not quantify food intake and it is possible that *A. tropicalis* inoculated flies displayed an altered feeding behavior resulting in reduced uptake of atrazine. The relationship between microbiome remodeling and chronic toxicity is therefore intriguing. Given that *A. tropicalis* can be cultured on atrazine without impacts on growth rates (Supplementary Fig. 8d), the depletion of *A. tropicalis* in the gut is unlikely to be a direct effect of chemical exposure—rather, an indirect effect of host metabolism on the competitive landscape of the gut—an example of toxicity and resilience that arises through the integration of host-microbiome metabolism. Given that *Acetobacter*, and not *Lactobacillus*, is sufficient to reduce atrazine toxicity to wildtype levels in gnotobiotic flies, this appears to be a disadvantageous situation for the host. We posit that increased abundance of *Acetobacter* is associated with improved outcomes for individuals—future studies will be needed to assess the merit of *Acetobacter* supplementation to protect against atrazine toxicity.

The fact that we were able to identify genes corresponding to most, but not all of the Atrazine degradation pathway (KEGG Map 00791) is also interesting, and highlights the challenge in the functional annotation of metabolic pathways, particularly in microbes. Whether this is a case of weak homology, or the presence of non-canonical enzymes able to functionally substitute for *atzD* is unclear. Engineering genetically tractable knockout bacterial strains would be useful to identify which genes enable atrazine detoxification.

## Materials and methods

### Fly husbandry

#### Conventional

This study was performed using the sequenced modENCODE *D. melanogaster* isogenic Oregon R BDSC strain 25211. Flies were reared and maintained at 25 °C on standard Drosophila medium (0.68% (w v^−1^) agar, 8.5% (w v^−1^) cornmeal, 0.4% (w v^−1^) active dry yeast, 0.024% (w v^−1^) sucrose, 0.028% (w v^−1^) potassium sodium tartrate, tetrahydrate, 0.004% (w v^−1^) CaCl2, 8.26% (v v^−1^) unsulphured dark molasses, 1.12% (w v^−1^) Tegosept, 0.01% (v v^−1^) ethanol, 0.017% (v v^−1^) propionic acid). To collect adults, flies were raised in 250 ml bottles containing 40 ml medium. Experiments including 16S microbiome and metagenomics, metabolomics, LC50 determination, RNA-sequencing, and the rescue experiment were conducted in the absence of Tegosept (see details below).

#### Age synchronization

To obtain adults for the treatment protocols we started by synchronizing embryos. Embryos were collected from three large embryo collection cages (3.5 in diameter × 6 in height). Each embryo cage was populated with 1 g of Oregon-R modENCODE flies, fitted with a molasses-agar (3.3% agar, 13% unsulfured molasses, 0.01% ethyl acetate, and 0.15% Tegosept) petri dish smeared with a 1−2 cm diameter circle of yeast paste (Fleischmann’s Baker’s Active Dry Yeast, 67% in water), was populated with 1 g of Oregon-R modENCODE flies and maintained at 25 °C under constant light conditions. Food plates were changed daily for up to one week. On the day of embryo collection, a fresh, yeasted, molasses-agar plate was attached for at least one hour, then replaced with another fresh, yeasted, molasses-agar plate for six hours. Embryos were harvested with 10−14 mL of sterile, 1xPBS buffer^35^, followed by 2−3 washes with 1×PBS. Thirty-two microliters of embryos were transferred to fly bottles containing standard fly food, one small Kimwipe, and active dry yeast pellets. The bottles were incubated at 25 °C, with constant light. On Day 9, bottles were cleared of any early-eclosing adults. On Day 10, adult flies (0−24 h post-eclosion) from multiple bottles were combined under brief CO_2_ anesthesia. Mixed-sex flies (*n* = 250) were counted, weighed, and collected into a large embryo collection cage. Subsequent cages were populated by equivalent weight. Cages were placed in a 25 °C incubator, with a 12 h/12 h light/dark cycle, at >60% relative humidity, for 4 days prior to exposure.

#### Germ-free

Embryos were collected as described above with the following modifications: bottles were cleared on Day 10 and adults collected on Day 11, as germ-free flies experience a developmental delay^36–38^ embryos were harvested with 10−12 mL of 95% ethanol, followed by a single wash with 5 mL 95% ethanol. Embryos were dechorionated by treatment with a solution of 50% bleach (3% sodium hypochlorite) for 2 min. In a Class IIa biosafety cabinet, embryos were washed three times with 5 mL sterile PBS. Using a wide-bore pipette tip ten microliters of settled embryos were transferred to sterile bottles containing autoclaved standard Drosophila medium and allowed to develop at 25 °C with >60% relative humidity, and 12 h/12 h light/dark cycle. Animals and tissues were immediately frozen in liquid nitrogen and stored at −80 °C for RNA preparations. We verified the status of our germ-free fly colony using a combination of culture and 16S PCR methods.

#### Atrazine rescue experiment

Germ free flies (0−24 h post-eclosion) were anesthetized by cooling in an empty, sterile fly bottle, on ice. Flies were counted on a bibulous paper-covered, inverted glass petri dish on ice. To avoid confounding behavioral effects of single-sex housing and sexing flies after death is unreliable, thirty AdMMF flies (15 female plus 15 male) were transferred into each sterile, glass shell vial containing 5 mL Yeast-Glucose (YG) fly food (10% w v^−1^ active dry yeast, 10% w v^−1^ glucose and 1.2% w v^−1^ Bacto-agar^39^; a strip of Whatman paper (2” by 1/2”); 2 mM atrazine in DMSO (0.25% v v^−1^), DMSO only, or YG food only; with and without *Acetobacter tropicalis* or two members of the family *Lactobacillaceae* (*L. brevis* and *L. mesenteroides*) (100 million CFU) in sterile 1xPBS) applied to the food surface. *Acetobacter tropicalis* was grown in Difco YPD broth (Yeast Extract-Peptone-Dextrose, BD Biosciences) at 30 °C to log phase. OD600 measurements were taken using a Nanodrop ND-1000 spectrophotometer, and the culture was pelleted and resuspended in sterile 1xPBS to an OD600 of 0.055 (107 cfu/µL). Ten microliters of resuspended culture were applied to the surface of sterile food in a glass shell vial. *L. brevis* and *L. mesenteroides* were grown in Difco Lactobacilli MRS (BD Biosciences) at 30 °C to log phase. OD600 measurements were taken using a Nanodrop ND-1000 spectrophotometer, and the culture was pelleted and resuspended in sterile 1 × PBS to an OD600 of 0.26 (10^7^ cfu/µL). Ten microliters of resuspended culture was applied to the surface of sterile food in a glass shell vial. Exposed and inoculated flies were transferred to fresh vials every 3−4 days during the 30-day exposure period. All materials were steam-sterilized at 121 °C in an autoclave. Glass shell vials containing YG food were sealed in an autoclave prior to sterilization and were only removed from the bag inside a biosafety cabinet; the vials were closed with sterile dense weave cellulose acetate plugs (Flugs^®^, Genesee Scientific). Experiments were maintained in closed plastic boxes in an incubator at 25 °C with >60% relative humidity, and on a cycle of 12 h light and 12 h dark. All procedures were performed in a Class IIa biosafety cabinet, except during transport between the incubator and the biosafety cabinet. All surfaces were sterilized with 70% ethanol before entering the biosafety cabinet.

#### Bacterial genomic DNA isolation

Bacteria were grown at 30−37 °C in an incubator/shaker at 150RPM under aerobic conditions. Cells were transferred to a 50 mL Falcon tube when the OD600 (Beckman DU640 spectrophotometer) was between 0.3 and 1 and pelleted. Genomic DNA was extracted using a modified phenol/chloroform method 8. Cell pellets were resuspended in 5 mL TE pH8.0; freshly prepared lysozyme (100 mg mL^−1^) was added to a final concentration of 3−5 mg mL^−1^ and incubated for 30−60 min at 37 °C. SDS (10% w v^−1^) was added to a final concentration of 0.5% v v^−1^; then 50 µL proteinase K (20 mg mL^−1^) was added and incubated at 56 °C for 1−3 h. The lysate was frozen in liquid nitrogen for 1 min then heated in 80 °C water bath for 3 min. The freeze-thaw cycle was repeated twice for a total of 3 cycles. After which 1.2 mL NaCl (4 M) and 1.2 mL CTAB/NaCl solution (10% CTAB w v^−1^ in 0.7 M NaCl, heated to 65 °C) were added and incubated for 10 min at 65 °C. The lysate was transferred to an Oak Ridge tube and an equal volume of phenol:chloroform:isoamyl alcohol (25:24:1 v v^−1^) pH8.0 was added. The solution was mixed by inversion, centrifuged for 10 min at 23,200 × *g* and the aqueous layer was transferred to a new Oak Ridge tube; then the phenol/chloroform extraction was repeated once. A final extraction was done with an equal volume of chloroform:isoamyl alcohol (24:1 v v^−1^), mixed by inversion, and centrifuged for 10 min at 23,200 × *g*. The aqueous layer was transferred to a 50 mL Falcon tube and the nucleic acids precipitated with 0.7 volumes of isopropanol at −20 °C for 1 h or overnight. DNA was pelleted for 20 min at 16,100 × *g*, washed with 70% ethanol, centrifuged for 10 min at 16,100 × *g*, air-dried, and resuspended in 200 µL 1× TE (pH8.0). RNA was removed by digestion with 10 µL of RNase I and incubated for 1 h at 37 °C. To remove the enzyme, we performed phenol/chloroform extractions and precipitated the DNA as above. The final pellet was resuspended in 100 µL 1× TE (pH8.0). The concentration was determined by Qubit and Nanodrop.

### Microbiome analyses

#### 16S rRNA sequencing

A DNA-free, sterile cotton swab was pre-wetted with HL3.1 Buffer^40^ then used to collect feces from the wall of a large embryo collection cage. The tip was placed into a PureLink Microbiome DNA Purification Kit (Invitrogen) bead tube, and the wooden handle was broken off. The supernatant was recovered and used for genomic DNA extraction. Genomic DNA was extracted from homogenized fly fecal samples collected daily after transfer of flies to fresh vials (Supplementary Fig. 9) using the Invitrogen PureLink Microbiome DNA Purification Kit according to the manufacturer’s instructions. PCR amplification of the V4 region of the 16S rRNA gene was performed using the protocol developed by the Earth Microbiome Project and described in^41^, using updated primers described by^42^. 16S sequencing was performed in a 20 µL reaction volume using 10X PCR Buffer without MgCl2 (1X); 50 mM MgCl2 (1.5 mM); RT­PCR grade water, 10 mM dNTP mix (0.3 mM each); 16S RNA gene primers (V4 region) 515 F (0.2 µM, 5′­GTGCCAGCMGCCGCGGTAA­3′) and 806R (0.2 µM, 5′­GGACTACHVGGGTWTCTAAT­3′­); Platinum Taq DNA polymerase (1U), fecal DNA prep (<500 ng). PCR was performed on ABI 9600 thermal cyclers using the following conditions: 94 °C for 4 min; 35 cycles of (94 °C for 45 s, 50 °C for 1 min, 72 °C for 1.5 min); 72 °C for 10 min; 4 °C hold. PCR clean-up was performed using AMPure XP beads. Sequencing libraries were made using the NEBNext Ultra DNA Library Prep Kit for Illumina Rev 3.0 with the following modifications: 5 µL PCR product plus 50.5 µL nuclease-free water were used for the end prep stage; cleanup of adaptor-ligated DNA proceeded without size selection; 9 cycles of PCR enrichment were performed using NEBNext Q5 Hot Start HiFi PCR Master Mix. Libraries were sequenced on an Illumina MiSeq using the 500-cycle (2×250nt) MiSeq Reagent Kit v2 (MS-102-2002, Illumina) according to the manufacturer’s instructions.

#### Metagenomics analyses

For metagenomic analysis, bacterial DNA was isolated from 20 adult 21-day old female fly guts. Whole flies were briefly anesthetized with CO_2_, washed once with 3% sodium hypochlorite and once with 70% ethanol, then dissected in sterile 1×PBS. The dissected guts were homogenized in 100uL 1×PBS buffer using a motorized disposable, autoclaved pestle. Following centrifugation at 1780 × *g* for 3 min, the supernatant was discarded and the pellet was resuspended in 100uL of QuickExtract DNA extraction solution (QE09050, Lucigen) and 1uL ReadyLyse Lysozyme Solution (30,000 U/uL, R1804, Lucigen). DNA was fragmented using NEBNext dsDNA Fragmentase (M0348S, New England BioLabs) and EDTA was removed with 1.5 volumes of AMPure XP (A63882, Beckman-Coulter Life Sciences) beads. Libraries were made using NEBNext Ultra DNA Library Prep Kit for Illumina (E7370S, New England BioLabs) and sequenced using a MiSeq v2 Reagent Kit (MS-102-2002, Illumina).

### Pesticide treatment

#### Atrazine and paraquat treatment for LC50 determination

For each treatment, 40 newly eclosed males and females (1:1) were transferred to fresh standard Drosophila medium containing vials and maintained at 25 °C for two days. To treat flies, two Kimwipes were folded into a square and put in the bottom of a glass shell vial (27.0 mm inner diameter (ID); 29 mm outer diameter (OD), 94 mm height). Kimwipes were saturated with 2 ml of the treatment solution, (10% sucrose solution w v^−1^ and 5% green food coloring v v^−1^, plus the treatment of interest). Although we did not measure atrazine levels in the frass, we confirmed the flies were eating based on green coloring of the abdomen. Therefore, differences in feeding behavior between dose groups could be a potential caveat in determining LC50 values. Harvesting time for adults varied by treatment. For paraquat treatment, four-day-old adults were fed 6−40 mM paraquat for up to 96 h. For atrazine treatment, four-day-old adults were fed 5−20 mM atrazine in 0.625−3.125% DMSO for 96 h. Following the treatment survival was assessed at seven timepoints up to 96 h.

#### Atrazine treatment for RNA isolation and sequencing

Adult flies (4 days post-eclosion) were transferred to clean, large embryo collection cages fitted with fresh food plates, with and without atrazine (Supplementary Fig. 9). Conventionally-reared four-day-old adults were fed 2 mM Atrazine in 0.25% DMSO for 72 h (3 days) in chemically defined food (CDF) 6/agar plates fitted onto collection cages without Tegosept. Germ-free four-day-old adults were fed 2 mM Atrazine in 0.25% DMSO for 72 h (3 days) in Yeast-Glucose (YG) fly food (10% w/v active dry yeast, 10% w/v glucose, and 1.2% Bacto-agar) without Tegosept 7. Following the treatment, adult flies were flash-frozen in liquid nitrogen and stored at −80 °C prior to RNA preparations. Four-day-old adults were fed 8 mM Paraquat for 72 h (3 days) in CDF 6 agar plates without Tegosept fitted onto collection cages. Following the treatment, adult flies were flash-frozen in liquid nitrogen and stored at −80 °C prior to RNA preparations. RNA was isolated using the RNeasy Plus Mini Kit (74134, QIAgen) and twenty gender-separated whole frozen flies (18−30 mg) per sample. The flies were homogenized using a Kontes pestle motor in an Eppendorf tube with 3 × 200 µL buffer RLT plus of the standard RNeasy Plus kit. The standard RNeasy Plus protocol was used with the following minor modifications; the lysate was centrifuged for 8 min at 12,000 RPM in an Eppendorf centrifuge 5415 and RNA was eluted with 35 µl of buffer. Prepared RNA was quantified using the Bioanalyzer Chip (RNA nano 6000) and the Qubit. The smaller of the two concentrations were used to calculate the amount of RNA needed for RNA library preparation (500 ng). The NEBNext Ultra Directional RNA Library Preparation Kit (E7420L, Illumina) together with NEBNext Poly(A) mRNA Magnetic Isolation Module (E7490L, Illumina) was used for RNA library preparation with the following modifications: Section 1.2 mRNA Isolation, Step 37, Fragmentation and Priming Total RNA, we decreased the incubation time from 15 to 5 min; Section 1.3 First Strand cDNA Synthesis, Step 2, we increased the incubation time from 15 to 50 min; for the size selection we used an insert size of 300−450 bp and a final library size of 400−550 bp; and finally in Section 1.9A, PCR library Enrichment, Step 2, we used 14 cycles for PCR cycling. RNA was stored at −80 °C prior to RNA sequencing. Strand-specific RNA-seq libraries were prepared from the treated and untreated samples using the NEBnext protocol. Samples were sequenced in biological triplicate. Libraries were sequenced on the Illumina HiSeq4000 platform using single-end 100 bp chemistry.

### Metabolomics

#### Conventionally raised flies

Fecal samples were collected by placing a standard glass microscope slide inside a large embryo collection cage, avoiding contact with the food plate. Fecal deposits were collected for one day, then stored at −80 °C until metabolomics analysis.

#### Germ-free raised flies

Flies were transferred from food vials to empty 2 mL cryovials by funnel and placed horizontally into the fly incubator at 25 °C for 1 h then returned to their original vials. Cryovials containing fecal samples were placed into −80 °C for storage until metabolomics analysis.

Nanoelectrospray ionization (nESI) direct-infusion mass spectrometry (DIMS)-based metabolomics and lipidomics were performed as previously reported^29^. Briefly, metabolites and lipids were extracted from fecal samples using a biphasic solvent extraction. Then, extracts were analyzed in positive and negative ionization modes using an Orbitrap Elite mass spectrometer (Thermo Fisher Scientific, Bremen, Germany) with a direct-infusion, chip-based nano-electrospray ionization source (Triversa, Advion Biosciences, Ithaca, NY, US).

### Computational analysis

#### 16S microbiome

QIIME 1.9.1 was used to demultiplex, quality filter, and join MiSeq libraries^43,44^. VSEARCH 2.4.1 was used to dereplicate, sort by abundance, remove single reads, and then to cluster at 99% similarity. VSEARCH was also used to check clusters for chimeras and construct an abundance table by mapping labeled reads to chimera-checked clusters^45–47^. Taxonomy was assigned to the centroid of each cluster using the Qiime script parallel_assign_taxonomy_uclust.py and the Greengenes database^48^.

Statistical analysis and visualization were performed in R using the packages Phyloseq, DESeq2, and ggplot2^49–51^. For bar plots, the 50 most abundant OTUs, representing 98.95% of the data, were agglomerated at the family and converted to relative abundances. Rarefaction curves were generated by subsampling reads (uniformly) from our 16S sequencing data with replacement and then computing species-level and agglomerated abundances using QIIME, as above. Operational taxonomic units were agglomerated as above. Ordination was performed and plotted with the Phyloseq package using the Principal Coordinates Analysis method and Bray−Curtis distances. Differential abundance analyses were performed at the OTU level using DESeq2 1.22.1. The effects of atrazine treatment over time were determined using a two-factor design (~Treatment + Time + Treatment:Time) and extracting the results for the interaction term. Differentially expressed clades were thresholded at a log fold change of 1.5 and an FDR adjusted *p* ≤ 0.01.

#### Metagenomics

To identify gut bacterial species, we used bowtie2 to remove reads that aligned to the Drosophila reference genome (Release 6)^52^, yeast (S288C, GCA _000146045), human (hg38), phiX (NC_001422), Illumina Tru-seq adapters, and RNA PCR primers. With non-bacterial reads removed we aligned to GenBank bacterial genomes and to known Drosophila gut microbes (e.g., Acetobacter aceti (CP014692)) and to our *Drosophila* gut microbe sequences^23–27,53^. More recently, we used Kraken 2 to analyze the sequence^54^.

#### Differential gene expression

Raw FASTQ files were aligned to the reference genome (Release 6)^52^ using STAR aligner 2.5.2b^55^ with default settings and up to 20 multiple alignments to produce BAM files. The HTSeq “htseq-count” command was run using the default “–nonunique none” option. Differential gene expression was determined using DESeq2 v1.9.34^51^ to with log_2_FoldChange cutoff −2 and 2, adjusted *p* ≤ 0.01.

#### Metabolomics

Direct-infusion mass spectrometry data were processed using DIMSpy as described previously^29^ (see Supporting Information for details). The processed feature intensity data matrices were used for statistical analyses. Mass-to-charge values of experimentally observed metabolic features were searched against the KEGG (https://www.genome.jp/kegg) database (modified to account for adducts), and all matches within a 5 ppm mass error tolerance were recorded^30^. Note that multiple annotations (e.g., isomeric compounds) could be observed for a single feature detected. Differential metabolite feature abundance was determined using DESeq2 v1.9.34 on the presence and absence transformed data after removal of the lowest quantile of DESeq2 estimated abundance (baseMean) and thresholding for adjusted *p* ≤ 0.001. The minimum observed value across all experiments was taken to be the bottom of the dynamic range for metabolic feature annotation. Non-observed metabolites were imputed as this minimum observed value to prevent infinite (or at least unbounded) fold-change values and to confine the presence-absence analysis to the empirical dynamic range.

#### Gene organ assignments

Gene lists from differential gene expression were used to associate genes with their organ assignment using modENCODE transcriptome data^56^ and the FlyAtlas 2 expression atlas^57^. Using specifically the FlyAtlas 2 expression microarray data for adult tissues, their seventeen dissected tissues were grouped into five categories: the digestive system comprises four tissues, the crop (analogous to the human stomach), salivary gland, midgut, and hindgut; metabolic processes include, carcass, fat body (analogous to human adipose tissue and liver) and malphigian tubules (analogous to the human kidney); the reproductive system in the male is comprised of the testis and accessory glands and in the female is comprised of the ovary, spermatheca (sperm-storage organs) and inseminated spermatheca; neural tissues include, head, brain, eye, and thoracic-abdominal ganglion; and the cardiovascular system is represented by the heart.

#### GO analysis

Gene Ontology biological annotations (*p* < 0.05) of differentially expressed genes (fold-change ≥1.5 and adjusted *p* ≤ 1E−06) were determined using ClueGO^58^ and visualized in Cytoscape^59^.

#### Integrative analysis

FlyScape (version 1.0.1) in Cytoscape (version 3.7.1) was used to visualize the metabolomics data in the context of metabolic reactions and transcriptional changes. Transcriptional changes in atrazine-treated female flies (72 h timepoint; *P* adjust <0.01) were combined with the union of all metabolomic changes after 24, 48, or 72 h of atrazine treatment and visualized in Flyscape^60^. The network was reduced to only include metabolites and genes from our input lists. Heatmaps were created using the gplots v3.0.1 R package.

### Statistics and reproducibility

R was used for all statistical analysis. Statistical significance was determined using statistical tests as indicated, including Student’s t-test and the Mann−Whitney U test. The number of animals and/or independent experiments are indicated in figure legends and the associated methods sections. Adjusted *p*-values are indicated as statistically significant where appropriate throughout the manuscript.

### Reporting summary

Further information on research design is available in the Nature Research Reporting Summary linked to this article.

## Supplementary information

Supplementary Information

Description of Additional Supplementary Files

Supplementary Data 1

Supplementary Data 2

Supplementary Data 3

Supplementary Data 4

Supplementary Data 5

Supplementary Data 6

Supplementary Data 7

Supplementary Data 8

Supplementary Data 9

Supplementary Data 10

Supplementary Data 11

Reporting Summary

## Data Availability

Strains are available from the BDSC public repositories. Complete 16S, RNAseq, and metagenomics datasets and all metadata are available from our BioProject accession number PRJNA718558. Data presented in Figs. 2a, b, f and 4a are available in Supplementary Data 11.
