# Supplementary material for: An integrated host-microbiome response to atrazine exposure mediates toxicity in Drosophila
Source: Commun Biol. 2021 Nov 24;4:1324. doi: 10.1038/s42003-021-02847-y (PMC8613235; doi:10.1038/s42003-021-02847-y)
Supplement: Supplementary file 1 — Supplementary Information [file 42003_2021_2847_MOESM1_ESM.pdf]

## Supplementary Figures

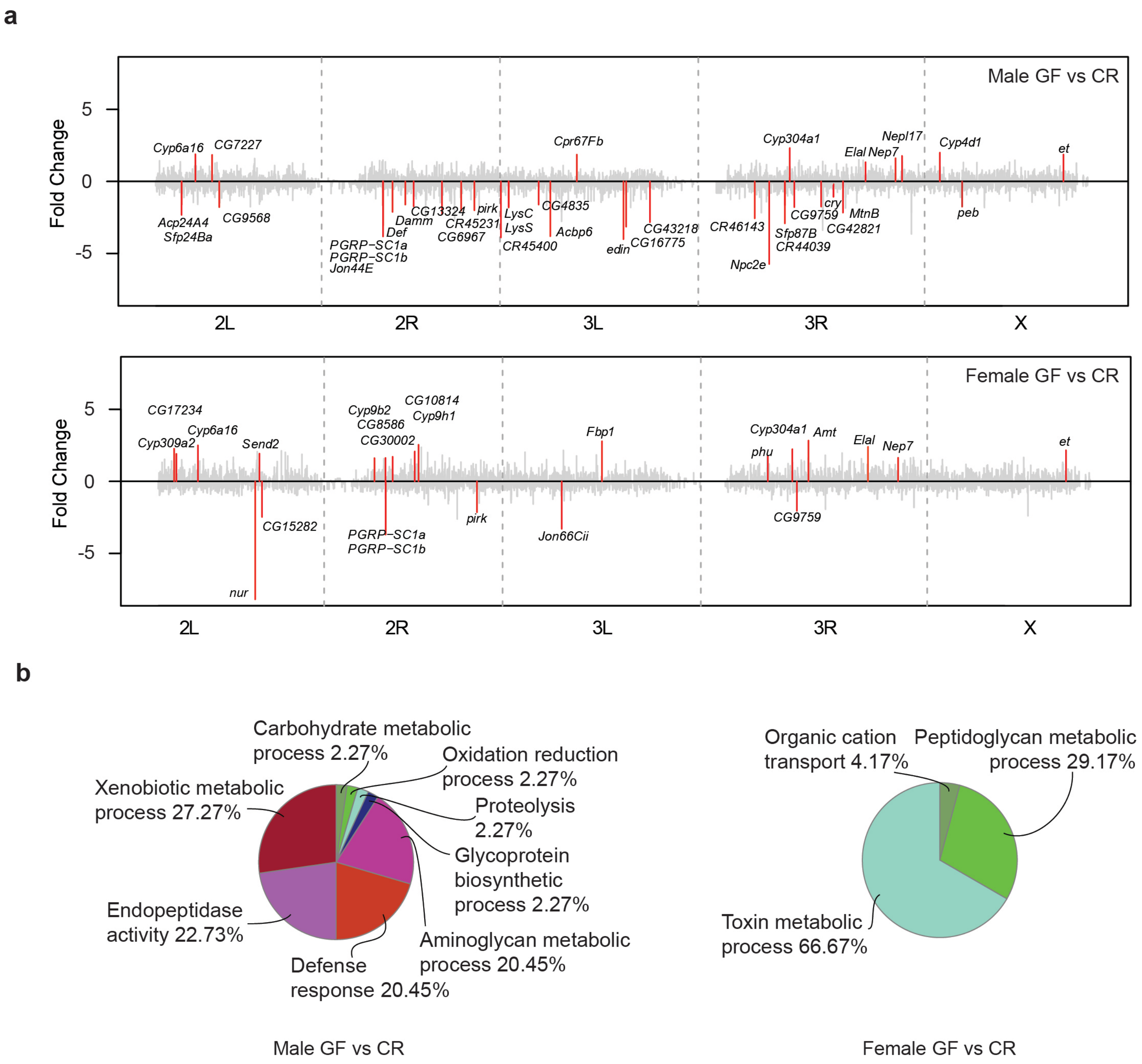


**Supplementary Figure 1. Male unexposed germfree vs conventional, Female unexposed germfree vs conventional spike plot and pathway annotations.** Transcriptional profiling of males and females raised conventionally or germ free. **a** Genes with differential expression between Conventionally reared (CR) and Germ Free reared (GF) animals. Genes are ordered on the chromosomes X, 2L, 2R, 3L, 3R and 4. All genes are shown in grey. **b** Gene Ontology analysis using ClueGO in Cytoscape ($p<0.05$) of genes differentially expressed ($FC \geq 1.5$ and $p \leq 1E\text{-}06$) after 72 hr in 2.0 mM atrazine treated conventional and germ-free reared flies compared to conventional and germ-free untreated control flies, respectively.

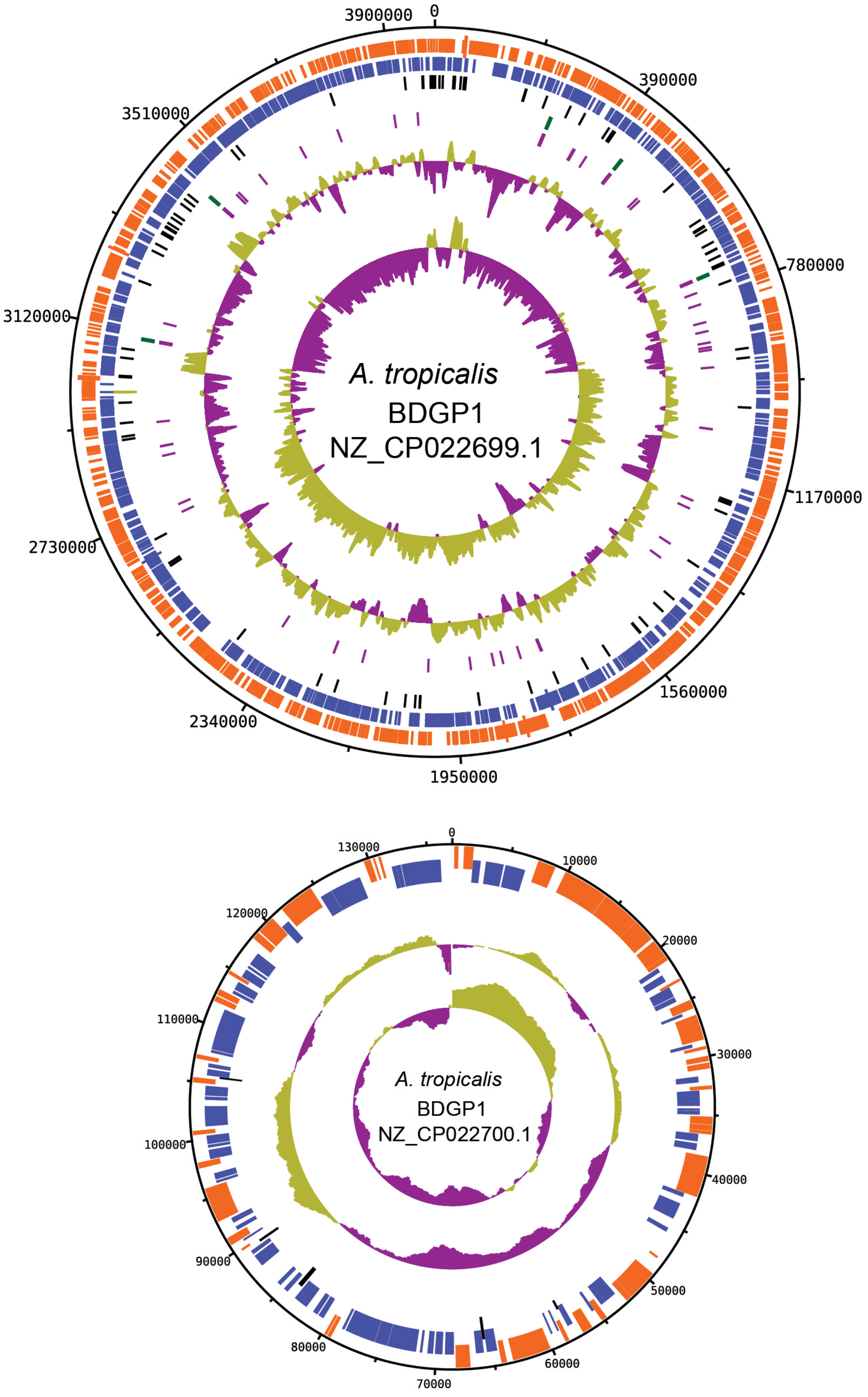


**Supplementary Figure 2. Circular plots for the A. tropicalis bacterial genome.** Plots were drawn using DNAPlotter and annotation files were generated by the NCBI Prokaryotic Genome Annotation Pipeline (PGAP). Plasmids are not drawn to scale relative to the chromosome. DNAPlotter tracks, beginning with the outermost track are: 1) positive strand genes (orange) 2) negative strand genes (blue) 3) pseudogenes (black) 4) rRNA (dark green) 5) tRNA (purple) 6) GC plot (purple/green) 7) GC skew (purple/green). The accession numbers are shown for each genome and plasmid assembly.

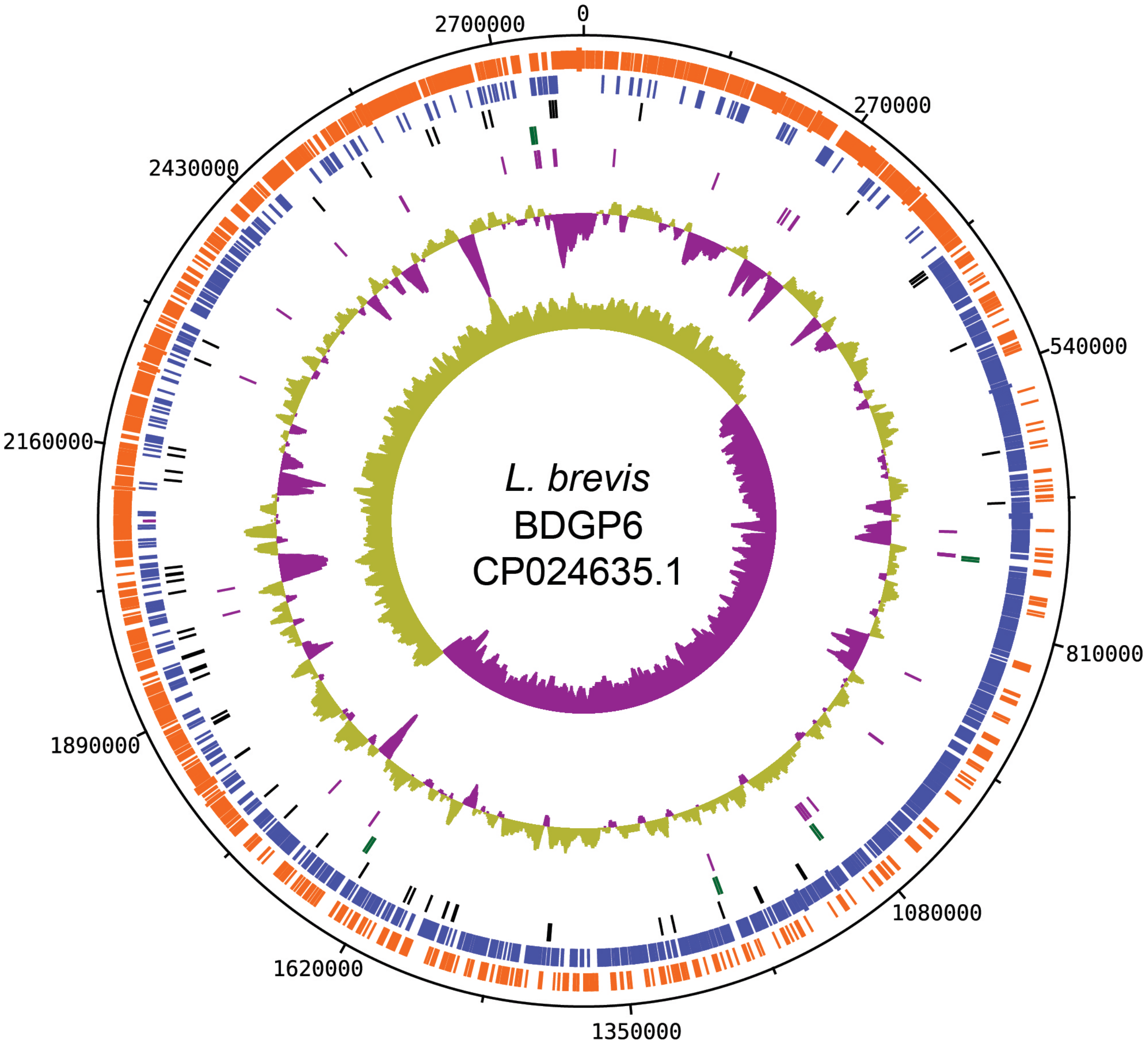


**Supplementary Figure 3. Circular plots for the L. brevis bacterial genome.** Plots were drawn using DNAPlotter and annotation files were generated by the NCBI Prokaryotic Genome Annotation Pipeline (PGAP). Plasmids are not drawn to scale relative to the chromosome. DNAPlotter tracks, beginning with the outermost track are: 1) positive strand genes (orange) 2) negative strand genes (blue) 3) pseudogenes (black) 4) rRNA (dark green) 5) tRNA (purple) 6) GC plot (purple/green) 7) GC skew (purple/green). The accession numbers are shown for each genome and plasmid assembly.

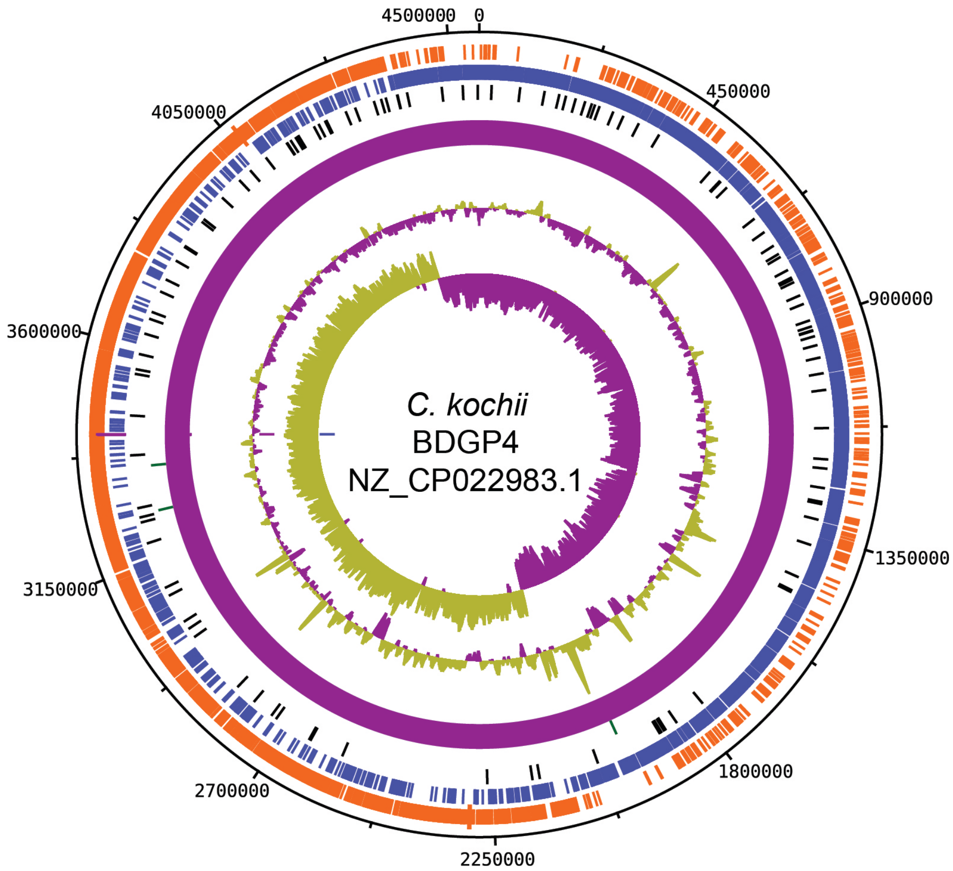


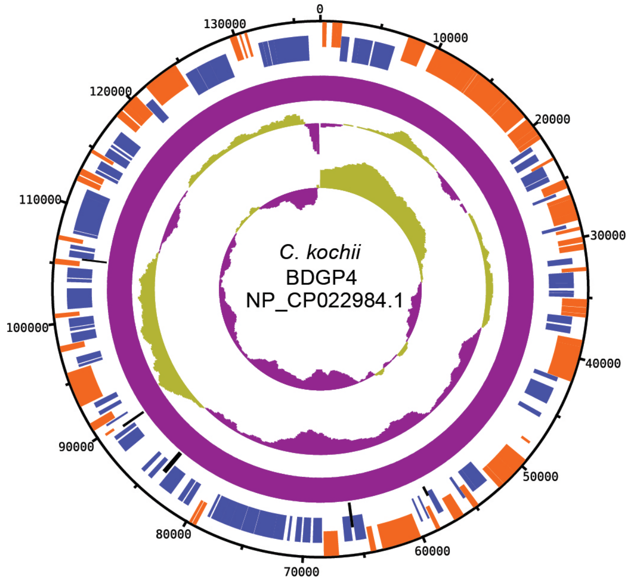


**Supplementary Figure 4. Circular plots for the C. kochii bacterial genome.** Plots were drawn using DNAPlotter and annotation files were generated by the NCBI Prokaryotic Genome Annotation Pipeline (PGAP). Plasmids are not drawn to scale relative to the chromosome. DNAPlotter tracks, beginning with the outermost track are: 1) positive strand genes (orange) 2) negative strand genes (blue) 3) pseudogenes (black) 4) rRNA (dark green) 5) tRNA (purple) 6) GC plot (purple/green) 7) GC skew (purple/green). The accession numbers are shown for each genome and plasmid assembly.

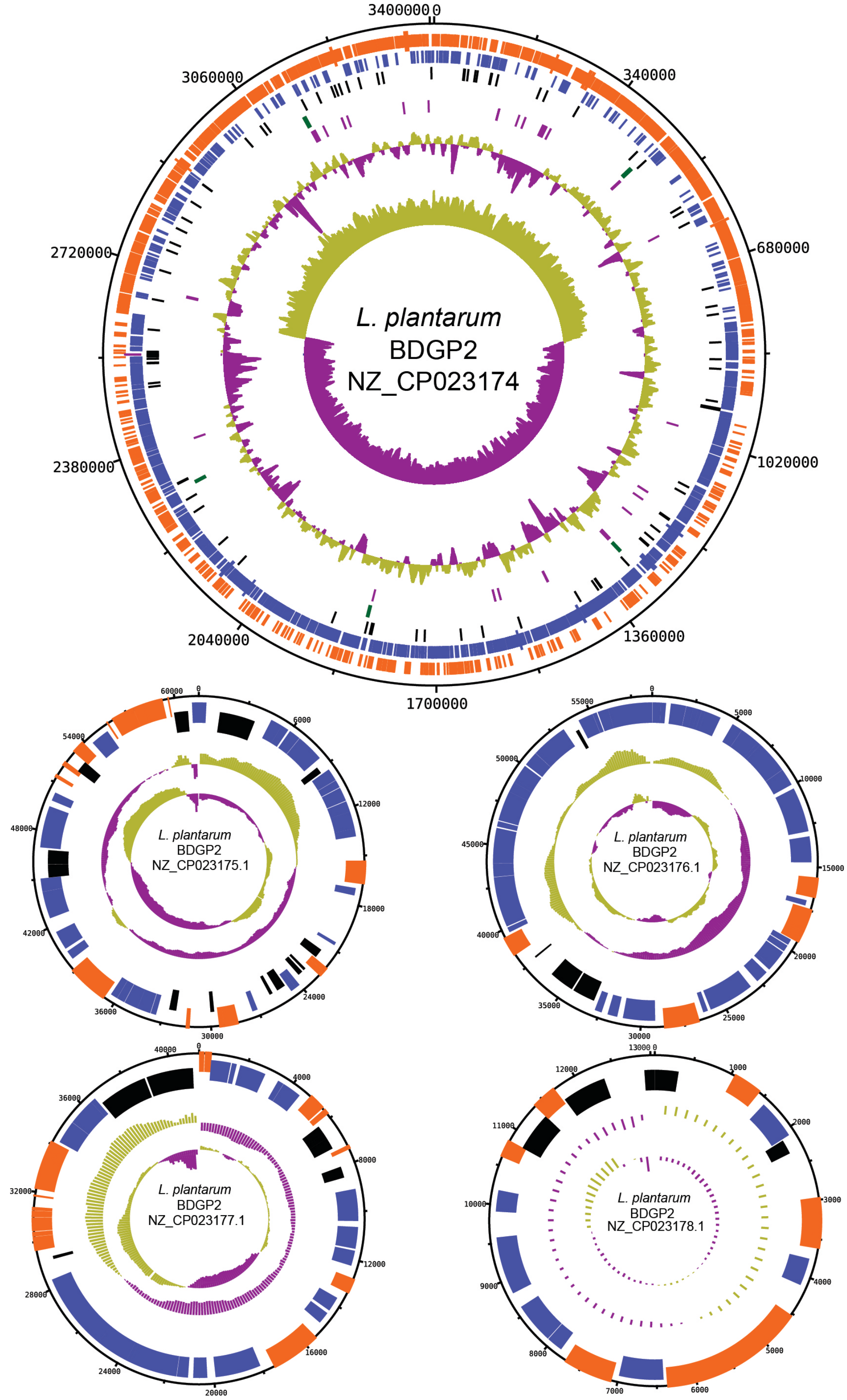


**Supplementary Figure 5. Circular plots for the L. plantarum bacterial genome.** Plots were drawn using DNAPlotter and annotation files were generated by the NCBI Prokaryotic Genome Annotation Pipeline (PGAP). Plasmids are not drawn to scale relative to the chromosome. DNAPlotter tracks, beginning with the outermost track are: 1) positive strand genes (orange) 2) negative strand genes (blue) 3) pseudogenes (black) 4) rRNA (dark green) 5) tRNA (purple) 6) GC plot (purple/green) 7) GC skew (purple/green). The accession numbers are shown for each genome and plasmid assembly.

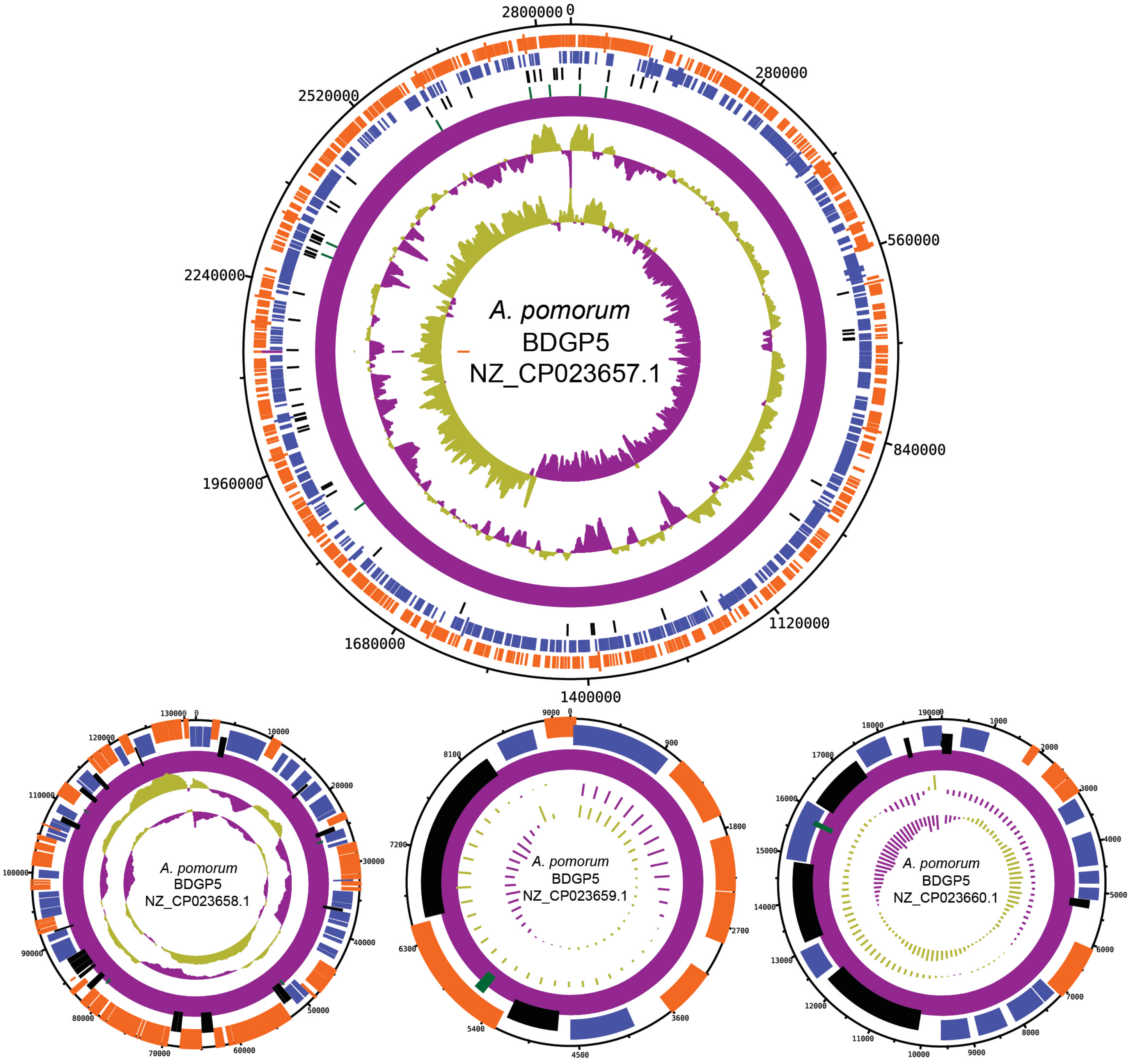


**Supplementary Figure 6. Circular plots for the A. pomorum bacterial genome.** Plots were drawn using DNAPlotter and annotation files were generated by the NCBI Prokaryotic Genome Annotation Pipeline (PGAP). Plasmids are not drawn to scale relative to the chromosome. DNAPlotter tracks, beginning with the outermost track are: 1) positive strand genes (orange) 2) negative strand genes (blue) 3) pseudogenes (black) 4) rRNA (dark green) 5) tRNA (purple) 6) GC plot (purple/green) 7) GC skew (purple/green). The accession numbers are shown for each genome and plasmid assembly.

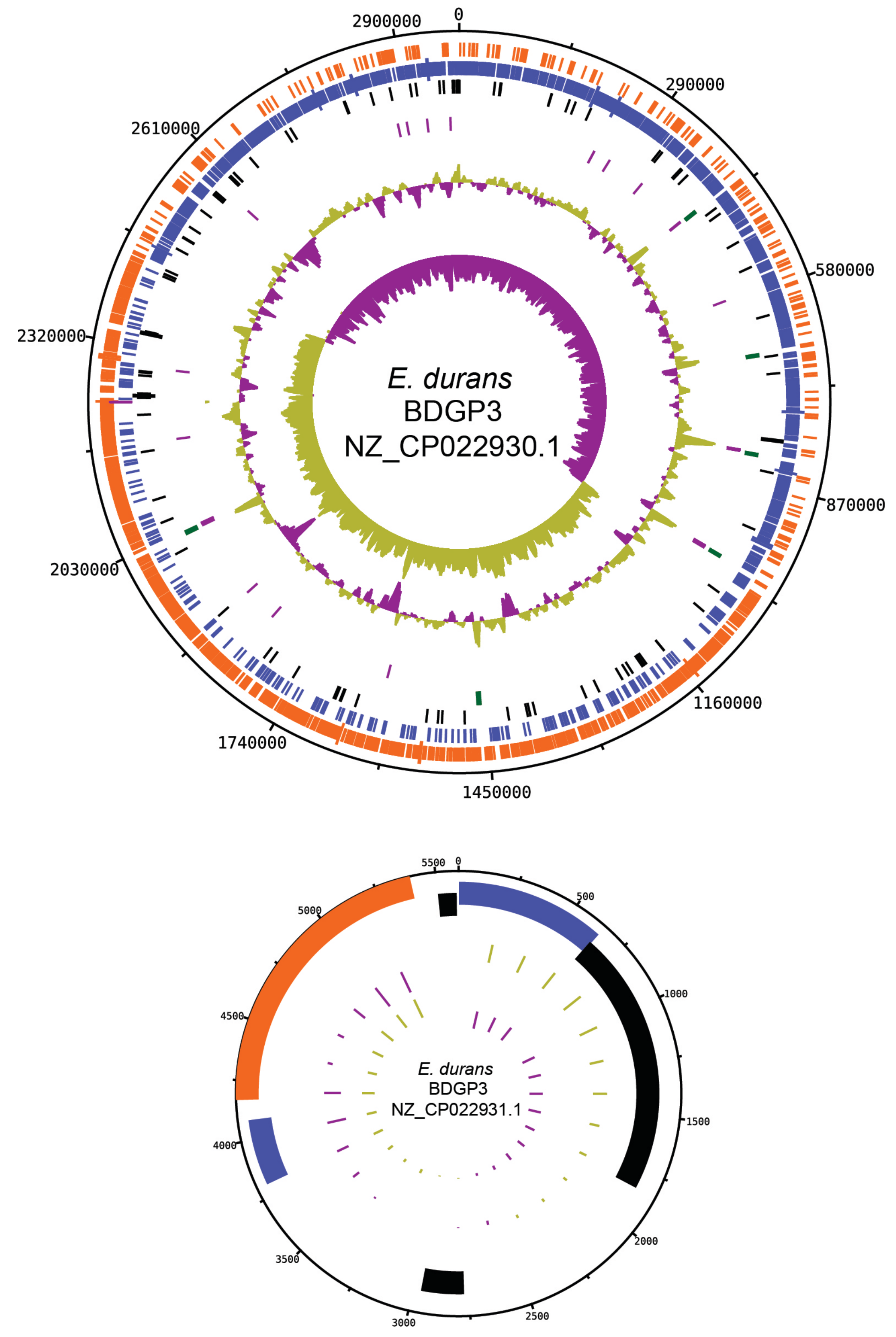


**Supplementary Figure 7. Circular plots for the E. durans bacterial genome.** Plots were drawn using DNAPlotter and annotation files were generated by the NCBI Prokaryotic Genome Annotation Pipeline (PGAP). Plasmids are not drawn to scale relative to the chromosome. DNAPlotter tracks, beginning with the outermost track are: 1) positive strand genes (orange) 2) negative strand genes (blue) 3) pseudogenes (black) 4) rRNA (dark green) 5) tRNA (purple) 6) GC plot (purple/green) 7) GC skew (purple/green). The accession numbers are shown for each genome and plasmid assembly.

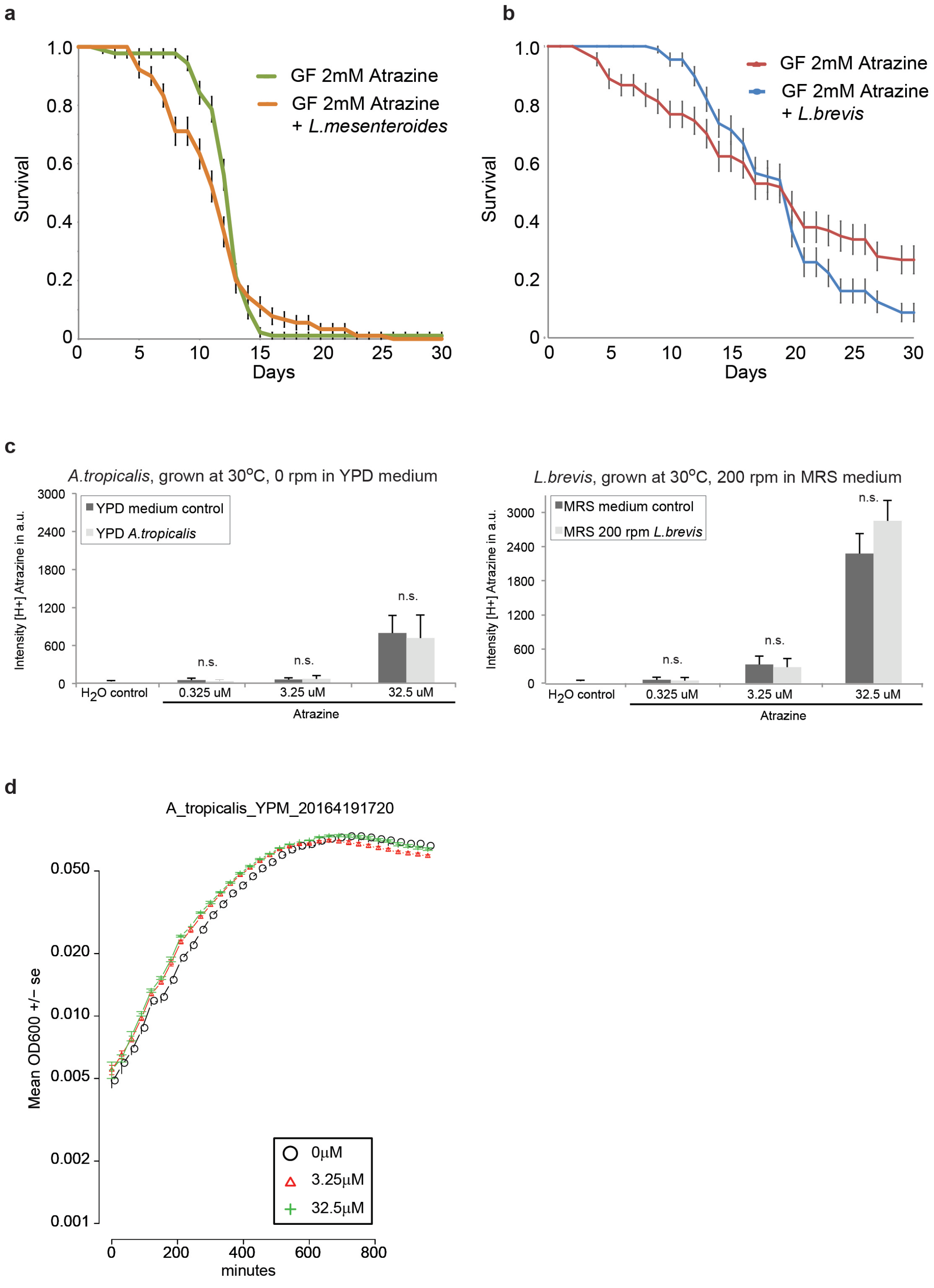


**Supplementary Figure 8. The relationship between microbes and atrazine toxicity. a** Survival curves of AdMMF GF flies exposed to 2mM atrazine or 2mM atrazine supplemented with *L.mesenteroides*. For each condition, we used 15 males and 15 females in triplicate aged 4-5 days post eclosion. Error bars indicate the standard deviation of the proportion of surviving flies. **b** Survival curves of AdMMF GF flies exposed to 2mM atrazine or 2mM atrazine supplemented with *L.brevis*. For each condition, we used 15 males and 15 females in triplicate aged 4-5 days post eclosion. Error bars indicate the standard deviation of the proportion of surviving flies. **c** Atrazine metabolism by *A. tropicalis* (left) and *L. brevis* (right) in culture media by MALDI as described (de Raad et al, Anal. Chem. 2017, 89, 11, 5818–5823). **d** Growth rate of *A. tropicalis* in YPM media (Yeast extract 5.0 g/L, Peptone 3.0 g/L Mannitol 25.0 g/L) in the presence of 0, 3.25 and 32.5 μM atrazine.

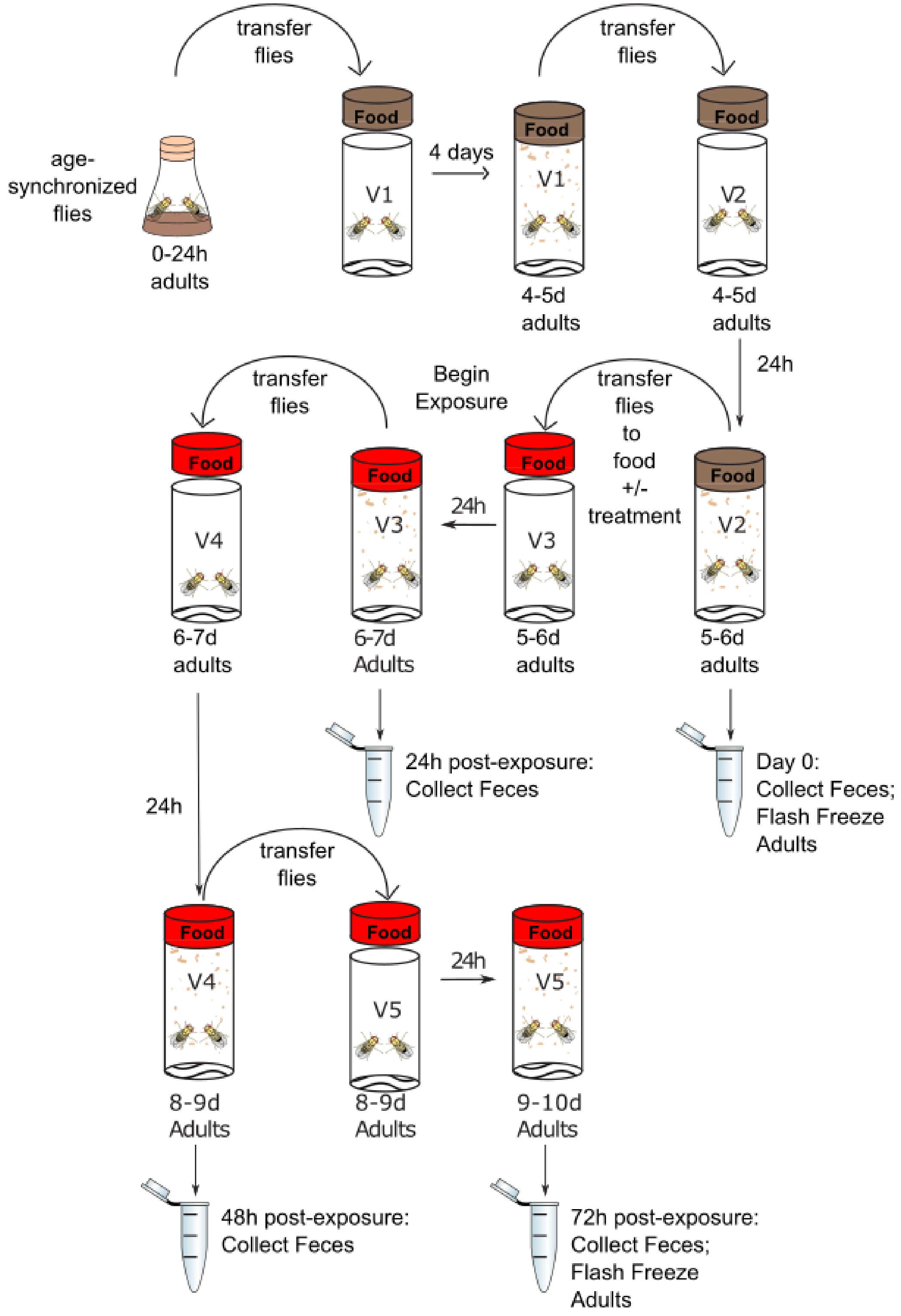


**Supplementary Figure 9. Example schematic of experimental sample generation for 16S fecal DNA sequencing and host RNA-sequencing.** Approximately, two-hundred and fifty AdMMF flies 0 - 24 hrs post-eclosion were transferred from bottles to cages (food in the diagram is colored brown) and aged 4 days to acclimate the flies to the cages and to allow their microbiomes to develop. We transferred the flies to new cages for 24 hrs and collected a zero time-point fecal sample and twenty flies (10 of each sex) for RNA-seq. After which we transferred the remaining flies to a new cage with food that included herbicide. Each subsequent transfer to new cages included treated food (food supplemented with herbicide in the diagram is colored red). Fecal samples were collected at 0, 24, 48 and 72 hrs. Flies for RNA-seq were collected at 0 and 72 hrs and sexed after being flash-frozen.
