# Supplementary material for: An integrated host-microbiome response to atrazine exposure mediates toxicity in Drosophila
Source: Commun Biol. 2021 Nov 24;4:1324. doi: 10.1038/s42003-021-02847-y (PMC8613235; doi:10.1038/s42003-021-02847-y)
Supplement: Supplementary file 2 — Description of Additional Supplementary Files [file 42003_2021_2847_MOESM2_ESM.pdf]

Description of Additional Supplementary Files

File name: Supplementary Data 1

Description: Public repository and metadata.

File name: Supplementary Data 2

Description: Microbiome genomes.

File name: Supplementary Data 3

Description: Male unexposed germfree vs conventional RNA-seq DeSeq Table

File name: Supplementary Data 4

Description: Female unexposed germfree vs conventional RNA-seq DeSeq Table

File name: Supplementary Data 5

Description: Male, 2mM atrazine exposure RNA-seq DeSeq Table

File name: Supplementary Data 6

Description: Female 2mM atrazine exposure RNA-seq DeSeq Table

File name: Supplementary Data 7

Description: Male germ free 2mM atrazine exposure RNA-seq DeSeq Table

File name: Supplementary Data 8

Description: Female germ free 2mM atrazine exposure RNA-seq DeSeq Table

File name: Supplementary Data 9

Description: Metabolomics - Raw data and annotations.

File name: Supplementary Data 10

Description: Metabolomics – Results DESEq2 statistical analysis.

File name: Supplementary Data 11

Description: Source data for Main Figures.
