# Supplementary material for: An integrated host-microbiome response to atrazine exposure mediates toxicity in Drosophila
Source: Commun Biol. 2021 Nov 24;4:1324. doi: 10.1038/s42003-021-02847-y (PMC8613235; doi:10.1038/s42003-021-02847-y)
Supplement: Supplementary file 4 — Supplementary Data 2 [file 42003_2021_2847_MOESM4_ESM.docx]

Supplementary Data File 2 Microbiome Genomes

1. Acetobacter tropicalis strain BDGP1 chromosome, complete genome

3,988,649 bp circular DNA Accession: NZ_CP022699.1

1a. Acetobacter tropicalis strain BDGP1 plasmid pAtBDGP1A, complete sequence

151,013 bp circular DNA Accession: NZ_CP022700.1

2. Acetobacter pomorum strain BDGP5 chromosome, complete genome

2,848,089 bp circular DNA Accession: NZ_CP023657.1

2a. Acetobacter pomorum strain BDGP5 plasmid pApBDGP5A, complete sequence

131,455 bp circular DNA Accession: NZ_CP023658.1

2b. Acetobacter pomorum strain BDGP5 plasmid pApBDGP5B, complete sequence

9,160 bp circular DNA Accession: NZ_CP023659.1

2c. Acetobacter pomorum strain BDGP5 plasmid pApBDGP5C, complete sequence

19,216 bp circular DNA Accession: NZ_CP023660.1

3. Levilactobacillus brevis strain BDGP6 chromosome, complete genome

2,785,111 bp circular DNA Accession: NZ_CP024635.1

4. Lactiplantibacillus plantarum strain BDGP2 chromosome, complete genome

3,407,160 bp circular DNA Accession: NZ_CP023174.

4d. Lactiplantibacillus plantarum strain BDGP2 plasmid pLtBDGP2D, complete sequence

13,055 bp circular DNA Accession: NZ_CP023178.1

4c. Lactiplantibacillus plantarum strain BDGP2 plasmid pLtBDGP2C, complete sequence

41,234 bp circular DNA Accession: NZ_CP023177.1

4b. Lactiplantibacillus plantarum strain BDGP2 plasmid pLtBDGP2B, complete sequence

58,689 bp circular DNA Accession: NZ_CP023176.1

4a. Lactiplantibacillus plantarum strain BDGP2 plasmid pLtBDGP2A, complete sequence

61,448 bp circular DNA Accession: NZ_CP023175.1

5. Levilactobacillus brevis strain BDGP6 chromosome, complete genome

2,785,111 bp circular DNA Accession: CP024635.1

6. Enterococcus durans strain BDGP3 chromosome, complete genome

2,983,334 bp circular DNA Accession: NZ_CP022930.1

6a. Enterococcus durans strain BDGP3 plasmid pEdBDGP3A, complete sequence

5,594 bp circular DNA Accession: NZ_CP022931.1

7. Cytobacillus kochii strain BDGP4 chromosome, complete genome

4,557,232 bp circular DNA Accession: NZ_CP022983.1

7a. Cytobacillus kochii strain BDGP4 plasmid pBkBDGP4A, complete sequence

137,143 bp circular DNA Accession: NZ_CP022984.1
