# Supplementary material for: An integrated host-microbiome response to atrazine exposure mediates toxicity in Drosophila
Source: Commun Biol. 2021 Nov 24;4:1324. doi: 10.1038/s42003-021-02847-y (PMC8613235; doi:10.1038/s42003-021-02847-y)
Supplement: Supplementary file 14 — Reporting Summary [file 42003_2021_2847_MOESM14_ESM.pdf]

nature research

Corresponding author(s): James B. Brown and Susan E. Celniker

Last updated by author(s): 2021/10/7

Reporting Summary

Nature Research wishes to improve the reproducibility of the work that we publish. This form provides structure for consistency and transparency in reporting. For further information on Nature Research policies, see our Editorial Policies and the Editorial Policy Checklist.

Statistics

For all statistical analyses, confirm that the following items are present in the figure legend, table legend, main text, or Methods section.

n/a	Confirmed	
☐	☒	The exact sample size (*n*) for each experimental group/condition, given as a discrete number and unit of measurement
☐	☒	A statement on whether measurements were taken from distinct samples or whether the same sample was measured repeatedly
☐	☒	The statistical test(s) used AND whether they are one- or two-sided<br>*Only common tests should be described solely by name; describe more complex techniques in the Methods section.*
☐	☒	A description of all covariates tested
☐	☒	A description of any assumptions or corrections, such as tests of normality and adjustment for multiple comparisons
☐	☒	A full description of the statistical parameters including central tendency (e.g. means) or other basic estimates (e.g. regression coefficient) AND variation (e.g. standard deviation) or associated estimates of uncertainty (e.g. confidence intervals)
☐	☒	For null hypothesis testing, the test statistic (e.g. *F*, *t*, *r*) with confidence intervals, effect sizes, degrees of freedom and *P* value noted<br>*Give P values as exact values whenever suitable.*
☒	☐	For Bayesian analysis, information on the choice of priors and Markov chain Monte Carlo settings
☒	☐	For hierarchical and complex designs, identification of the appropriate level for tests and full reporting of outcomes
☒	☐	Estimates of effect sizes (e.g. Cohen's *d*, Pearson's *r*), indicating how they were calculated

Our web collection on statistics for biologists contains articles on many of the points above.

Software and code

Policy information about availability of computer code

Data collection	For 16S sequencing: Amplicons were sequenced on an Illumina MiSeq using the 500 cycle (2x250nt) MiSeq Reagent Kit v2 according to manufacturer's instructions.<br>For metagenomics: Libraries were made using NEBNext Ultra DNA Library Prep Kit for Illumina (E7370S, New England BioLabs) and sequenced using a MiSeq v2 Reagent Kit (MS-102-2002, Illumina).<br>For RNA-seq: Samples were sequenced in biological triplicate. Libraries were sequenced on the Illumina HiSeq4000 platform using single-end 100 bp chemistry.<br>Metabolomics: The metabolomes of fecal samples were analyzed using nano-electrospray ionization - direct infusion mass spectrometry (nESI-DIMS).
Data analysis	For 16S sequencing: QIIME 1.9.1 was used to analyze the data. Statistical analysis and visualization were performed in R using the packages Phyloseq, DESeq2, and ggplot2. Differential abundance analyses were performed at the OTU level using DESeq2 1.22.1. Ordination was performed and plotted with the Phyloseq package using the Principlal Coordinates Analysis method and Bray-Curtis distances. Convex hulls were added to the plot using ggConvexHull.<br>For RNA-seq: Raw FASTQ files were aligned to the reference genome (Release 6) using STAR aligner 2.5.2b with default settings and up to 20 multiple alignments to produce BAM files. The HTSeq "htseq-count" command was run using the default "--nonunique none" option. Differential gene expression was determined using DESeq2 v1.9.34.<br>For metabolomics: Data processing was done using the Galaxy online platform using the selected ion monitoring (SIM) stitching algorithm (see supporting information for details). The processed data matrices were used for statistical analyses. Differential metabolomics abundance was determined using DESeq2 v1.9.34.

For manuscripts utilizing custom algorithms or software that are central to the research but not yet described in published literature, software must be made available to editors and reviewers. We strongly encourage code deposition in a community repository (e.g. GitHub). See the Nature Research guidelines for submitting code & software for further information.

Data

Policy information about availability of data

All manuscripts must include a data availability statement. This statement should provide the following information, where applicable:

- Accession codes, unique identifiers, or web links for publicly available datasets
- A list of figures that have associated raw data
- A description of any restrictions on data availability

All sequencing data has been deposited to NCBI under Study SRP312722 and BioProject PRJNA718558.
All other relevant data supporting the key findings in this study are available within the article or its Supplementary Data Files or from the corresponding author upon reasonable request.

Field-specific reporting

Please select the one below that is the best fit for your research. If you are not sure, read the appropriate sections before making your selection.

[x] Life sciences [ ] Behavioural & social sciences [ ] Ecological, evolutionary & environmental sciences

For a reference copy of the document with all sections, see nature.com/documents/nr-reporting-summary-flat.pdf

Life sciences study design

All studies must disclose on these points even when the disclosure is negative.

Sample size	The number of flies used is sufficient to detect pesticide toxicity based on previous studies using the same resource. Permutation tests were used to ascertain significance of differentially expressed genes and metabolites between treated and control groups or between germ-free and conventionally reared flies. (See Methods)
Data exclusions	Samples that failed to sequence (<4000 reads per sample) were excluded. This exclusion criterion was pre-chosen, based on prior experience.
Replication	All experiments were conducted in triplicate.
Randomization	The experiments were randomized. Flies were randomly assigned to the study groups. The analysis order of fly fecal samples was fully randomized for the 16-S and GC-MS analysis including process blank controls and retention index calibrating compound daily.
Blinding	The investigators were not blinded for the toxicity studies. For metagenomic, 16S sequencing and GC-MS analysis, the analysis was blinded.

Reporting for specific materials, systems and methods

We require information from authors about some types of materials, experimental systems and methods used in many studies. Here, indicate whether each material, system or method listed is relevant to your study. If you are not sure if a list item applies to your research, read the appropriate section before selecting a response.

Materials & experimental systems

n/a	Involved in the study	
[x]	[ ]	Antibodies
[x]	[ ]	Eukaryotic cell lines
[x]	[ ]	Palaeontology and archaeology
[ ]	[x]	Animals and other organisms
[x]	[ ]	Human research participants
[x]	[ ]	Clinical data
[x]	[ ]	Dual use research of concern

Methods

n/a	Involved in the study	
[x]	[ ]	ChIP-seq
[x]	[ ]	Flow cytometry
[x]	[ ]	MRI-based neuroimaging

Animals and other organisms

Policy information about studies involving animals; ARRIVE guidelines recommended for reporting animal research

Laboratory animals	Drosophila melanogaster Oregon-R-modENCODE, 4-7 days of age after eclosion
Wild animals	n/a
Field-collected samples	n/a
Ethics oversight	n/a

Note that full information on the approval of the study protocol must also be provided in the manuscript.
